# Mapping a research-advocacy-policy agenda on human rights and albinism: a mixed methods project

**DOI:** 10.1186/s12939-023-02064-5

**Published:** 2024-01-02

**Authors:** Sheryl Reimer-Kirkham, Barbara Astle, Ikponwosa Ero, Lori Beaman, Bonny Ibhawoh, Elvis Imafidon, Richard Sawatzky, Wisdom Tettey, Meghann Buyco, Emma Strobell

**Affiliations:** 1 Trinity Western University, 22500 University Drive, Langley, BC V2Y 1Y Canada; 2 African Albinism Network, c/o UTSS, PO Box 32837, Dar es Salaam, Tanzania; 3 University of Ottawa, 75 Laurier Ave. E, Ottawa, ON K1N 6N5 Canada; 4 McMaster University, 1280 Main St W, Hamilton, ON L8S 4L8 Canada; 5 SOAS University of London, 10 Thornhaugh St, London, WC1H 0XG UK; 6 University of Toronto, 1265 Military Trail, Scarborough, ON M1C 1A4 Canada

**Keywords:** Albinism, Human rights, Meta-narrative review, Priority-setting survey, Social determinants of health, Spiritual beliefs, African ontology, Explanatory framework, Mixed methods

## Abstract

**Background:**

Persons with albinism face challenges to their wellbeing, safety, and security, ranging from vision impairment and skin cancer to stigma and discrimination. In some regions, they also face human rights atrocities including mutilation and murder. Research on human rights and albinism is a relatively new field that has gained momentum since the United Nations appointment of an Independent Expert on the enjoyment of human rights by persons with albinism. In this paper, we present the results of a mixed methods study undertaken to identify priorities for research, advocacy, and policy on albinism and human rights.

**Methods:**

The first component was a synthesis of peer-reviewed and grey literatures at the nexus of albinism, spiritual/cultural beliefs and practices, and human rights. We then conducted a priority-setting survey, informed by Delphi methods, on extant knowledge-practice gaps and research, advocacy, and policy priorities. Inclusion criteria included demonstrated expertise in the field (e.g., peer-reviewed publications, funded research), membership on national or international associations, or advocacy (civil society organizations) of more than 2 years in albinism and human rights. Thereafter, we gathered leading researchers, policy-makers, and civil society stakeholders for a Roundtable to gain consensus on these priorities.

**Results:**

Access to skin and vision care, and education were not deemed high priority for research, likely because the evidence supporting the need for these is well established. However, they were priorities for advocacy and policy: what is needed is mobilization of this evidence through advocacy and implementation of such services (policy). Other social determinants of health (rurality, poverty, and gender equality) are present as subtext in the findings, more so than priorities for research, advocacy, or policy, despite their preponderance in the lives of persons with albinism. Research was prioritized on stigma and discrimination; advocacy; and witchcraft, but with some differentiation between Global North and Global South priorities. Priorities for research, advocacy, and policy vary in keeping with the explanatory frameworks at play, including how harmful practices and witchcraft are viewed.

**Conclusions:**

The lived experience of albinism is profoundly shaped by the social determinants of health (SDOH). Threats to the security and well-being of persons with albinism should be viewed through a human rights lens that encompasses the explanatory frameworks at play.

**Supplementary Information:**

The online version contains supplementary material available at 10.1186/s12939-023-02064-5.

## Background

Research on the welfare and security of persons with albinism (PWA), especially through a human rights lens, is a relatively new field. Worldwide, persons with the rare genetic condition of albinism (in particular, those with Oculocutaneous Albinism Type 2), lack melanin or pigmentation in their skin, hair, and eyes, and hence are vulnerable to vision impairment and skin cancer. Along with these health concerns, they may face stigma and discrimination, social isolation, and lack of access to health and social services (such as disease prevention with sunscreen and skin cancer screening). In some regions (predominantly in Sub-Saharan Africa, but also in Asia), deeply rooted spiritual/cultural beliefs persist about evil, misfortune, and curses, such that PWA are ostracized and excluded. In more extreme cases, PWA face human rights atrocities including mutilation and murder as their body parts are reportedly used for witchcraft purposes and economic gain through trafficking [1–4]. Civil society organizations such as *Under the Same Sun (UTSS), Standing Voice, Amnesty International*, and local communities and governments responded to these human rights violations with child protection legislation, public education, and advocacy. One outcome of this advocacy was the United Nations Human Rights Commission appointment in 2015 of Ms. Ikponwosa Ero as the first Independent Expert on the enjoyment of human rights by persons with albinism. The mandate of the Independent Expert is to report and advise on human rights, and since its inception, the priority issues have been witchcraft-related harmful practices, discrimination, disability, health, and women and children. A research-advocacy-policy network was formed by AUTHOR and AUTHOR, at the invitation of the United Nations (UN) Independent Expert in 2016 to support the new mandate with research. The network was formed to understand more fully the scope of the threats to human rights, the underlying mechanisms, and possible social responses. In this paper, we present the results of a mixed methods research project undertaken to identify priorities for research, advocacy, and policy on albinism and human rights.

### Background and literature review

A range of health-related spiritual/cultural meanings contribute to the stigma and discrimination experienced by PWA [5–10]. In conjunction with the more extreme versions of dehumanizing attributions, various forms of violence have been reported, including intimate partner and sexual violence, infanticide, threats, mutilation, and murder. The albinism literature reports on harmful practices associated with the manifestation of belief in witchcraft, including the idea that the body parts of PWA can bring success and good luck, though fewer studies explicate this relationship in detail [1, 11]. Reimer-Kirkham et al.’s [12] scoping review revealed the under-researched area of how broader social structures influence PWA disproportionately, resulting in health and social inequities. Poverty and lack of access to housing, health services, and education were noted in many of the articles, though often without explicit language of the social determinants of health (SDOH) that are vital to understanding the relationship between social factors and poor health. Along with the SDOH, a human rights lens is needed to offer deeper analysis and broader recommendations to address the atrocities perpetrated against PWA. Without a human rights framing, the causes, effects, and solutions related to the albinism, spiritual/cultural, and health nexus remain under-theorized and not well understood [12].

## Methods

**Fig. 1:**
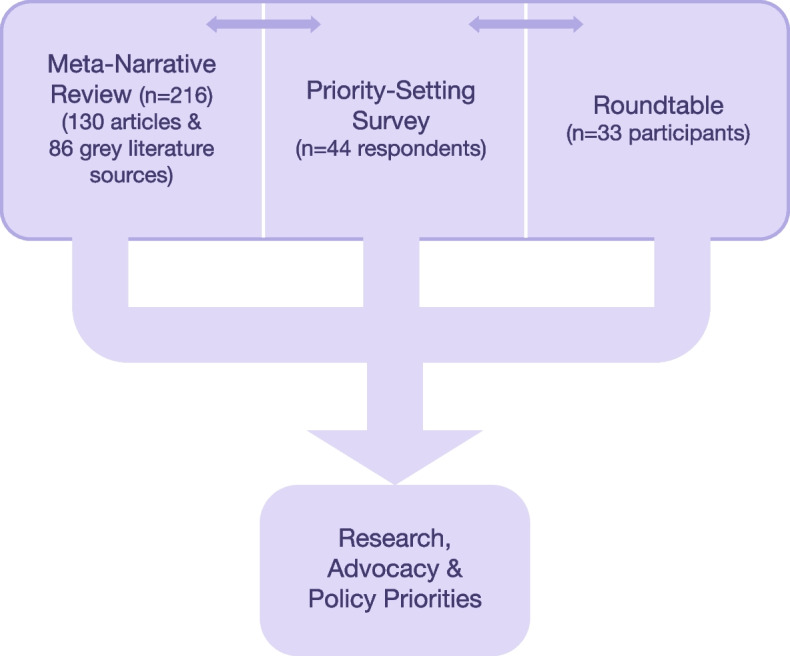
Mixed methods components and sources of data (*n* = 293 sources of data)

The purpose of the mixed methods project was to foster (i) evidence-informed policy and human rights advocacy through the development of an international, interdisciplinary research-advocacy-policy network and (ii) a prioritized research agenda on albinism, spiritual/cultural beliefs and practices, and human rights. As the project progressed, priorities for advocacy and policy were also noted, in part because advocacy itself was a research priority. Phase I was a synthesis of peer-reviewed and grey literatures on albinism, spiritual/cultural beliefs and practices, and human rights. Phase II involved a priority-setting survey, informed by Delphi methods, to elicit expert opinion on current research underway, extant knowledge-practice gaps, and initial consensus on research priorities [13]. In Phase III we gathered leading researchers, policy-makers, and civil society stakeholders for a Roundtable (see Fig. 1). All three sources of data informed one another (both sequentially and concurrently) and were synthesized to arrive at an overall conclusion.

### Phase I: meta-narrative review

Our synthesis was guided by meta-narrative review [14, 15] approaches that are designed to create “meta-narratives” of evidence on complex (and perhaps contested) issues as the basis for creating an overarching “storyline” [15, 16]. We broadly followed Greenhalgh et al.’s [14] 6 steps to meta-narrative review, using the Realist and Meta-narrative Evidence Synthesis: Evolving Standards (RAMESES) as a guide [15].

#### Planning phase

Based on our consultation with project co-lead Ero and other experts, and our scoping review [12], we developed the following overarching review question: What perspectives, policies, and practices best protect the human rights of persons with albinism? The question focused on three intersecting concepts: albinism; human rights; spiritual/cultural beliefs and practices. We worked in consultation throughout each phase to ensure relevance and appropriateness of the methods taken [14].

#### Searching phase

The search phase occurred between January 2018 to October 2020. The albinism concept included search terms of *albinism*, albino*, achrom*, *oculocutaneous*, and hypopigment*. The spiritual/cultural practices concept included search terms of cultur*, spirit*, *diversity*, *witchcraft*, *beliefs*, and *worldview*. Search terms for the human rights concept included human right*, civil right*, and *security*. The final search string was constructed based on the intersection of the searches for these three concepts. Relevant databases (e.g., Academic Search Complete, ALTA Religion Database, JSTOR, PsychINFO, and Social Science Citation Index) were searched. Backward searches and forward searches were conducted with the most relevant articles. Grey literature sources were identified by searching Google Scholar and key stakeholder websites that serve as repositories of albinism-related resources. No time filter was applied given a general paucity of peer-reviewed literature. Results were merged using Endnote software and duplicates were removed [17].

To summarize the current state of knowledge (academic literature, grey literature) at the juncture of albinism, spiritual/cultural practices, and human rights, inclusion and exclusion criteria focused the systematic review, with the inclusion criteria of: a) must substantively relate to all 3 concepts (albinism; spiritual/cultural practices; and human rights), b) must relate to policy, and c) must be either academic peer-reviewed literature or grey literature written for or by a recognized organization. Exclusion criteria were not in English, French, or Portuguese.

**Fig. 2:**
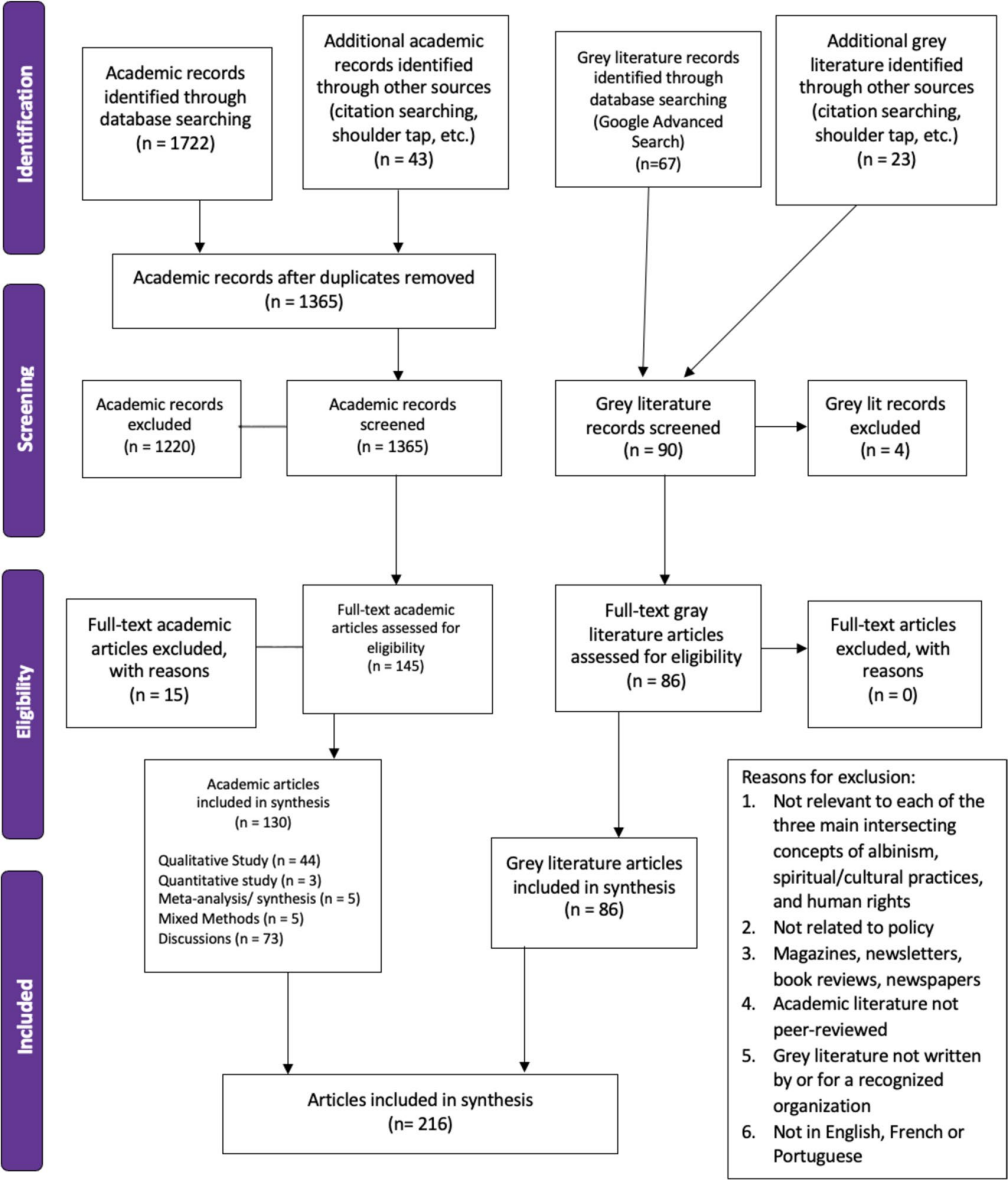
PRISMA flow diagram [18]

After duplicates were removed, 1,365 articles were double screened. Incongruencies were resolved by team discussion. A total of 216 articles met the criteria for this synthesis, consisting of 130 academic articles and 86 grey literature sources (see Fig. 2).

#### Mapping phase

All sources were uploaded into NVivo™, a qualitative data analysis software (see Supplemental Materials: Matrix) [19]. We developed a codebook as an evaluative framework with extraction questions, for example, about location, method, sample, purpose, main findings, assumptions or explanatory frameworks, and recommendations provided. Initially, two researchers applied the extraction questions to five articles and compared their findings to establish interrater reliability. Coding was completed by four trained research assistants. We read across the data sources for additional themes and gaps in the literature as the basis for constructing the storylines.

Methods distribution for the academic literature is as follows: qualitative (*n* = 44), quantitative (*n* = 3), mixed (*n* = 5), systematic (*n* = 5) and discussion articles (*n* = 73). These studies represented health (nursing, medicine, social work, and public health), social sciences (anthropology, sociology, psychology, disability studies, cultural studies, media studies, communication, geography, archeology), education, arts and humanities (critical arts, literature, and philosophy, culture, religion), and law (legal, finances, and banking). The articles ranged from 1946 to 2020 with 67 articles published within the past 5 years. The grey literature, the articles consisted of government documents, national non-governmental organization reports and articles, and international organization reports. The years ranged between 2009 to 2020 with 60 articles published within the past 5 years. The majority of both academic and grey literatures focused their research and reports on African countries (Nigeria, Tanzania, South Africa, Kenya, Malawi, Uganda, Zimbabwe, Cameroon, and Ghana) but there were articles that met the inclusion criteria where research occurred in USA, Jamaica, Brazil, UK, Australia, Canada, and Dominican Republic. Of the articles in the academic literature, only 64 articles had first authors from Africa. Only 18 articles reported research in high-income countries (USA, Canada, Australia, UK, and Germany), but 64 had lead authors from high-income countries located primarily in the northern hemisphere. In the grey literature, 24 articles were authored by international organizations.

#### Appraising phase

Each source was appraised by two researchers for rigour and relevancy to determine their strengths and limitations; where there was discrepancy between the reviewers a project lead facilitated consensus. The academic literature sources were appraised using the Joanna Briggs Institute appraisal tools [20] as well as the Mixed Method Appraisal Tool [21]. Grey literature articles were appraised using the Public Health Ontario guide [22]. To speak to the meaningfulness of including grey literature, we added the significance section of the AACODS (Authority, Accuracy, Coverage, Objectivity, Date, Significance) checklist [23].

#### Synthesis phase

The synthesis phase entailed a meta-narrative approach that involved aggregation, contextualization, and interpretation to create storylines that thread through the various perspectives. The core team met regularly to discuss the emerging storylines about how research on albinism and human rights has unfolded and how research questions are framed as to priorities and underlying assumptions. This phase involved reading and re-reading the data [15].

#### Recommending phase

The recommending phase evolved out of the survey and the Roundtable, both reported in this manuscript.

### Phase II: priority-setting survey

**Fig. 3:**
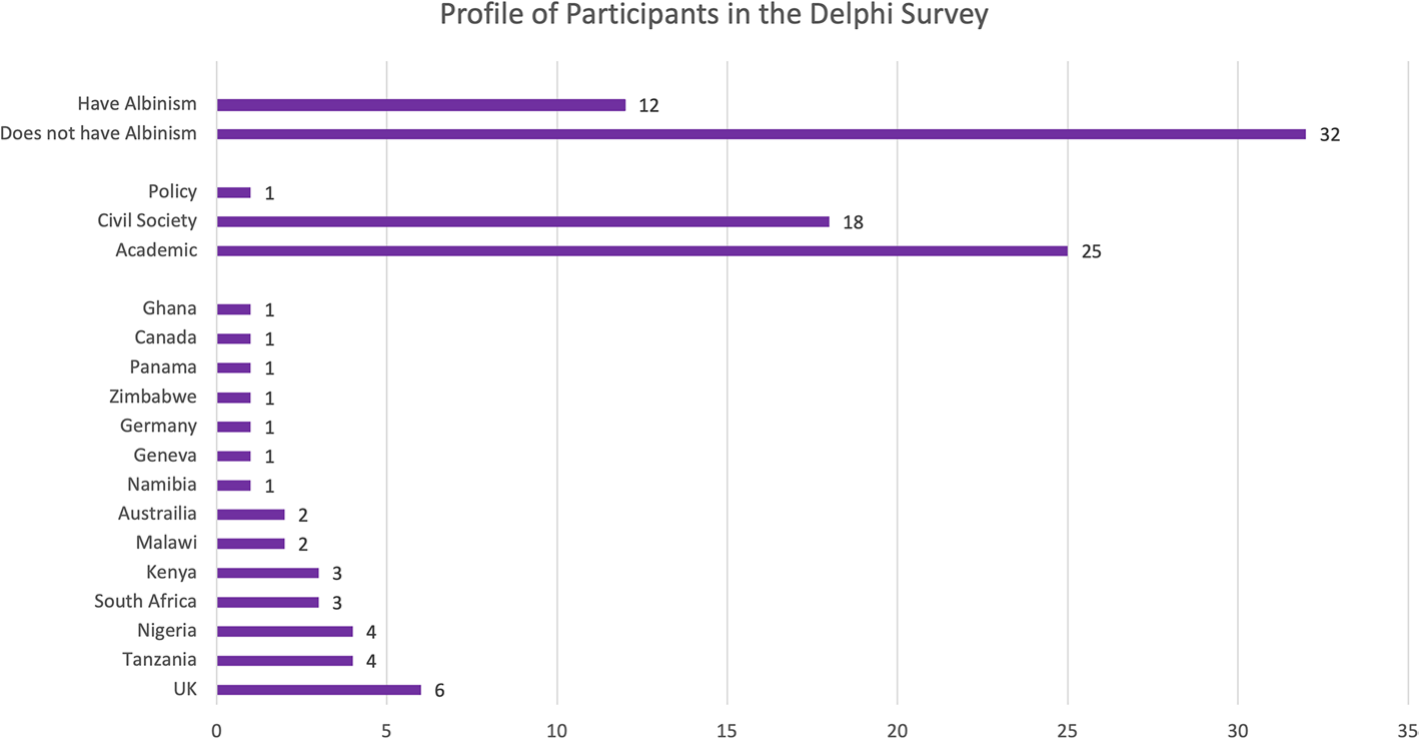
Profile of participants in survey

An online, two-round priority-setting survey, informed by Delphi methods, was used to gather input from experts about priorities on albinism and human rights [24]. Sampling involved identifying 120 experts in the field of albinism research. Inclusion criteria were: demonstrated expertise in the field (e.g., peer-reviewed publications funded research), or membership in national or international albinism associations or advocacy (civil society organizations) of more than 2 years in albinism and human rights. Forty-four respondents completed either one or both rounds (see Fig. 3). About 30 percent were PWA and 50 percent were from the Global South (predominantly Africa). Many self-identified as both academics and advocates.

Round 1 posed broad questions to identify priorities in research, advocacy, and policy in the realm of albinism and human rights, such as: *What research questions and advocacy strategies are needed in regard to albinism, spiritual/cultural practices, and human rights? What evidence-informed policy is required?* Content analysis was used to synthesize the written responses [25], and together with data from the literature synthesis, we generated 155 items as possible priorities, grouped as advocacy strategies, policy initiatives, and research topics (See Supplemental Materials: Survey Items). Round 2 had the respondents rating each item under specific clusters (in no specific order) under the three areas of Research, Advocacy Strategies, and Policy Initiatives using a 5-point Likert scale: Essential; Very Important; Important; Less Important and Unimportant. Then the calculated scores of agreements and disagreement from the rated items were ranked from highest to lowest within each cluster. Areas of agreement and disagreement were surfaced with this method as an important step in establishing priorities [26]. See Table 1 for the ranking of the two highest items (Essential) and two lowest (Unimportant) items for each cluster.

**Table 1:** Priority-setting survey results

**Order of Priority**	**Top Two High Scores**	**Scores**	**Bottom Two Low Scores**	**Scores**
**Advocacy strategies**				
**Cluster #1:** Local Advocacy Strategies	Improve skin care	97.4 15^a^	Use literature 26	43.6
	Access to education	94.9 11	Use theatre (plays) 24	42.1
**Cluster #2:** International advocacy Strategies	Strategies originating from international NGOs 1	94.8	Financial assistance from international community 4	82.1
	Cooperation to prevent cross-border crimes 6	92.3	Lobby for openness to PWA seeking asylum 5	53.9
**Cluster #3:** Avenues of Influence	Primary teachers in schools 42	89.5	Seek alliances with supportive politicians 34	74.4
	Make central albinism support groups 36	87.2	Witchdoctors and traditional healers 37	66.7
**Cluster #4:** Intersectoral Advocacy Strategies^b^	Strategic alliances among NGOs 8	94.9	Alleviate poverty 9	64.1
	Promote Albinism Awareness Day 10	69.2		
**Policy initiatives**				
**Cluster #1:** Health and Social Policy	Free eye and skin care 46	92.3	Poverty reduction 52	74.4
	Integrated classrooms 50	92.3	Policy specific to older PWA 53	53.8
**Cluster #2:** Policing	Strengthen local policing 73	89.2	Sting operations to intercept trafficking 74	76.3
	Special prosecution task force 72	78.9	Pursue justice in internation courts 71	46.5
**Cluster #3:** Legislation and Regulation	Child protection policies 65	84.6	Ban TV and radio programs that stereotype 69	59.0
	Regulation of witchcraft practitioners 67	82.1	Regulate Nollywood 70	56.4
**Cluster #4:** Professional Groups	Create National Office for Albinism 54	87.2	Create Local Officer for Albinism 55	59.0
	Increase number of teaching assistants 57	76.9	Increase number of vision specialist teachers 58	56.4
**Research priorities**				
**Cluster #1:** Stigma, myths, beliefs	How to end discrimination 81	88.9	Experience of older PWA 79	50.0
	Ways to dismantle public health myths 82	86.1	Death myth 80	44.4
**Cluster #2:** Research about Advocacy	Effectiveness of albinism advocacy strategies 149	88.6	Social dynamics with and within the disability movement 154	62.9
	How to integrate voices of PWA 153	85.3	Human rights - comparing albinism with other disabilities 151	57.1
**Cluster #3:** Witchcraft	Best ways to engage witchdoctors 134	85.3	How economies of religion relate to killings 128	60.0
	Impact of witchcraft on PWA 124	80.0	Human sacrifice of PWA 123	58.8
**Cluster #4:** Psychological Wellbeing	Resilience 85	83.3	Impact of trauma 86	75.0
	Social change in Africa 88	75.0	Trauma-informed care and policy 87	75.0
**Cluster #5:** Economic and Social Rights	Educating employers 116	83.3	Variability of service 111	52.8
	Accommodations in the workplace 115	72.2	Effect of agrarian to global market economies 109	40.0
**Cluster #6:** Health and Social Care	Improve providers’ knowledge & practices 98	82.9	Genetic testing 91	44.4
	Local specialist low-vision care 94	72.2	Cultural views of wearing glasses 95	33.3
**Cluster #7:** Family	How to support families 106	83.3	Families’ knowledge of genetics 104	58.3
	Lives of children with albinism 103	67.6	Families and safety	58.3
			going to school 105	
**Cluster #8:** Law	Human trafficking139	74.3	Applicability of international criminal law 137	60.0
	Effectiveness of safehouses 142	74.3	Legislation needed to grant asylum 140	45.7
**Cluster #9:** Religion and Church	How to engage faith leaders 120	69.4	African ontologies 118	69.4
	Role of faith communities 119	63.9	Everyday religion in Africa 117	54.3
**Cluster #10:** Media and Communication^b^	Impact of media on stereotypes 146	65.7	Media bias in Europe and U.S. 145	45.7
	Role of Nollywood films 147	45.7		

^a^Refers to the survey item number. For details, see Supplementary Material: Survey Items

^b^This cluster has three items

### Phase III: roundtable

The third data source came from a 2-day Roundtable convened by the UN Independent Expert on albinism and the research team with a representative group of 33 scholars, policy-makers, and civil society stakeholders. The Roundtable was held on 20 and 21 September 2018 in Geneva, as a side event at the thirty-ninth session of the Human Rights Council. The Roundtable served as a forum for bringing the voices of activists into the processes of research, and for prioritizing areas for research, advocacy and policy (Full Report: A/HRC/40/62/ADD.2) [27].

## Results: diffracting and weaving storylines

The central theme of the results centers on priorities for addressing the intersectoral and intersecting dimensions of human rights of PWA. Although we initially intended to establish consensus on research priorities, the mix of concordant and discordant storylines made us realize that more helpful than consensus would be an openness to the possibilities gained from understanding the underlying dynamics shaping these priorities. We structure our presentation of findings by summarizing two synthesized storylines: first, that the lived experience of albinism is profoundly shaped by the SDOH; and second, that threats to the security and well-being of PWA should be viewed through a human rights lens that extends to the explanatory frameworks (causal narratives). Supporting evidence from the literature review, the priority-setting survey, and the Roundtable elucidates (dis/con)cordances within each storyline.

### Storyline 1: the lived experience of albinism and the social determinants of health in need of human rights protection

The synthesized data portrays the lived experience of albinism as fraught with challenges, many of which trace back to a lack of access to the SDOH. Although not always employing the language of SDOH, strong consensus exists—across the academic and grey literature and expert stakeholders, between the Global North and the Global South, and increasingly shared by sectors and disciplines—regarding the key features that must be addressed for the security and well-being of PWA. In particular, strong concordance exists that the more proximal SDOH (those that act directly or almost directly to cause disease) are the most impactful in the day-to-day lived experience of PWA [28]. Though not cited as frequently, there is moderate consensus about more distal SDOH (those further back in the causal chain and acting via intermediary causes) and their influence on the lived experience of PWA. The survey rankings of priorities for advocacy, policy and research were integrated into the interpretation of this storyline, as was the Roundtable discussion. See Table 1 for the survey ranking of the two highest items (Essential) and two lowest (Unimportant) items. See Table 2 for literature citations regarding lived experiences of PWA and the SDOH.

**Table 2:** Storyline 1 – lived experiences of PWA and the SDOH

**Domain**	**Academic Literature References (*n* = 130)**	**Grey Literature References (*n* = 86)**
**Proximal**		
Visual impairment; risk for skin damage/cancer. May limit outdoor activities due to (i) not having protective clothing and eyewear, and (ii) because of fear of violence *n* = 104 (48%)	[6–8, 10–12, 29–80] *n* = 58 (45%)	[3, 27, 81–124] *n* = 46 (53%)
Access to Education: Schooling and learning are impacted by (i) visual impairment, (ii) access to quality education with accommodations, (iii) bullying, and (iv) separation from family (if in boarding schools) *n* = 122 (56%)	[5–8, 10, 11, 29, 30, 32, 33, 35–37, 39–45, 47–50, 53–56, 58–62, 64–66, 68–71, 73, 75, 77–80, 125–152] *n* = 74 (57%)	[27, 81, 82, 84–86, 88–95, 97–102, 104–107, 110, 111, 114–116, 118–124, 153–164] *n* = 48 (56%)
Community-level Social Inclusion/Exclusion: (i) lack of knowledge about albinism, (ii) stigma and discrimination, (iii) abandonment; (iv) emotional and physical abuse. Service providers, community members, employers *n* = 176 (81%)	[1, 2, 5–12, 29–32, 35–47, 49, 51–57, 59–74, 76–80, 125–128, 130–134, 137–144, 146–152, 165–185] *n* = 101 (78%)	[3, 27, 81–92, 94, 95, 97–102, 105–116, 118–124, 153–159, 161–164, 186–207] *n* = 74 (86%)
**Distal**		
Place and Geography: (i) prevalence of albinism, (ii) amount of sun exposure, (iii) explanatory systems at play (i.e., African ontology), (iv) location of attacks, (v) regulation of traditional healers, (vi) rural/remote/low resource areas with limited access to services and policing/ security, and (vii) other SDOH *n* = 90 (42%)	[1, 2, 5, 7, 8, 10–12, 31–33, 37–39, 42, 44, 47, 49, 51, 56, 60, 61, 63, 64, 67, 70, 74–76, 78–80, 126, 128, 134, 139, 145, 146, 149–152, 167, 171, 173, 174, 180–183, 185, 208, 209] *n* = 53 (41%)	[3, 27, 81, 86, 89, 90, 92, 94, 95, 98, 99, 101, 106, 107, 111, 113, 115, 118–120, 122, 124, 153, 157, 159, 160, 164, 187, 190, 193, 194, 197, 198, 201, 202, 204, 206] *n* = 37 (43%)
Poverty and Employment: (i) access to income, (ii) subsistence living, (iii) employment conditions can involve damaging sun exposure and lack of knowledge about albinism, and (iv) access to transportation *n* = 73 (34%)	[1, 2, 5–7, 10, 12, 30, 33, 36, 37, 41, 42, 44, 46, 47, 54, 60–62, 68–70, 73–75, 78–80, 125, 129, 130, 134, 150, 152, 167, 171, 184] *n* = 38 (29%)	[3, 27, 82, 87, 89–91, 94, 95, 97–99, 101, 102, 104, 106, 111, 112, 114–116, 118–122, 124, 155, 160, 163, 164, 188, 193, 194, 206] *n* = 35 (41%)
Gender Equality *n* = 17 (8%)	[10, 36, 43, 44, 51, 69, 79, 80, 148, 177] *n* = 10 (8%)	[96, 103, 114, 124, 162, 163, 210] *n* = 7 (8%)

The numbering in the table corresponds to the References

#### I. Proximal social determinants of health (SDOH)

The three most cited proximal SDOH are presented here from more proximal (i.e., immediate, direct) to less proximal.

- **Access to vision and skin care.** Strong consensus exists across academic and grey literatures and expert stakeholders that a fundamental route to addressing the SDOH for PWA involves access to health services, especially vision and skin care. Of 130 academic articles included, 44% (*n* = 58) noted access to vision and skin care [36, 55, 58]. Fifty-three percent (*n* = 46) of grey literature also did so [85, 116, 121]. The literature synthesis established that access is more of an issue in rural areas [61, 80, 102, 118, 122], and in countries with weaker health systems [41, 56, 97]. For PWA living in rural areas in lower resource countries, they may be reliant on services provided by civil society organizations [11, 12, 206].
  Lack of access to vision and skin care has far-reaching consequences, as PWA are reported to limit outdoor activities due to not having protective clothing and eyewear, which in turn affects employment, quality of life, and social inclusion [64, 86, 101, 105, 152]. Something as basic as providing free and accessible sunscreen is flagged as a priority by many sources [30, 32, 35, 58]. Similarly, the lack of vision care impacts access to education (e.g., not being able to see the blackboard [6, 31, 81, 154]. The grey literature substantiates these concerns, and in addition portrays civil society organizations as active in advocacy for and provision of vision and skin care (e.g., through mobile clinics) [33, 39].
  The *survey* respondents did not rate access to skin and vision care as a high priority for research, but did so for policy (the item “free eye and skin care was tied as #1 for policy initiatives in the Health and Social Policy cluster) and advocacy (the item “advocacy to improve skin care for PWA” was rated #1 among priorities for local advocacy strategies). See Table 1. This observation is consistent with the strong research evidence on the need for access to vision and skin care, and the need for advocacy and policy to advance implementation of these services. In the words of one survey respondent: “*…a considerable amount of data is already available on vision and skin care; therefore more a matter of good change management and evaluation of progress”*. The Global South puts priority on comprehensive healthcare (beyond vision and skin).
  The *Roundtable* consultation confirmed access to vision and skin care as a pre-requisite to moving forward. In the words of one attendee: “*Low vision and lack of pigment are the only two real difference between PWA and non-PWA. Every waking moment is affected by low-vision - this element must be accounted for in our research and inform us.*”
  Across data sources (i.e., literature, survey, and Roundtable), access to vision and skin care is portrayed as a foundational contributor to the lived experience of PWA. Where PWA have stable access, they are able to engage in education, employment, and everyday activities without undue concern.
- **Access to education.** A significant amount of research-derived evidence cites access to quality education for children with albinism (56%, *n* = 122), including (i) the negative impact of lack of teachers’ knowledge about the required accommodations for vision impairment [43, 60, 68, 101, 118]; (ii) bullying by classmates and teachers [5, 7, 89, 119, 133, 142, 150, 159]; (iii) the benefits of integrated (inclusive) education, given the effects of separation from family for children in boarding schools [36, 78, 89, 149]; and (iv) issues around safety while at school, and while coming and going from school [69, 80, 124]. The grey literature provides guidance for policy for albinism-friendly schools [115, 121, 122], with an emphasis that secure schools should be part of a government response [89, 101].
  The *survey* ranked “advocacy to improve access to education for PWA” as #2 for local advocacy strategies, and policy initiatives for education were also rated highly (#2 in the clusters for Health and Social Policy, and Professional Groups). Yet, as with access to vision and skin care, access to education as a research priority did not rate in the top 2. Here a discrepancy between Global South and Global North ratings adds important nuance. Two items rated highly by the Global South (but not the Global North) were: “research on how vision impairment impedes education, confidence, independence and social inclusion” and “research to identify factors that impede PWA from realizing rights to education”. This discordance suggests that local, contextualized research can substantiate the need for government response for improved educational services.
  Education, specifically public education about albinism as an advocacy strategy and policy initiative, was a recurrent theme at the *Roundtable*. In particular, civil society organizations and policy stakeholders emphasized that their organizations’ mandates and activities give priority to education.
- **Stigma and discrimination countered by social support and social inclusion.** The literature has a strong concordant theme to do with identifying and addressing the stigma and discrimination experienced by PWA. Illustrating the centrality of this theme, a majority of the included sources name discrimination and stigma (academic = 78%, grey = 86%). Abundant evidence exists in the academic and grey literature on the impact of discrimination and stigma on PWA, including feelings of inferiority, worries about abandonment, social isolation and withdrawal because of stigma and discrimination [10, 45, 82, 94, 152, 172, 188]. Underpinning discrimination and stigma is a lack of knowledge about the causes of albinism, which results in the perpetuation of myths and stereotypes. Discussed in detail in the second storyline below, stigma and discrimination extend to the violence experienced by many PWA. On balance, the academic literature places stronger emphasis on stigma, social support, and schooling, whereas the grey literature places stronger emphasis on safety, trauma, and violence.
  The literature revealed the mental health toll of stigma and discrimination (*n* = 65), but few studies have mental health as the primary focus [5, 68]. Mental health interventions tend toward social support more than professional services. The literature inclusion criteria of a human rights-related scholarship may well mean that some mental health literature is not included in this synthesis.
  Social support from family, community members and service providers is presented in the literature as vitally important in countering the psychosocial impact of discrimination, stigma, trauma and violence. In the case of albinism in Africa, family and community support takes on heightened meaning, as women and children with albinism are often abandoned by their husbands and extended families, placing them at greater risk for poverty and violence [64, 79, 80, 84, 95]. Some literature referred to an implicit duty for families and PWA to be brave and promote positive attitudes [100]. Some sources report on various media and public engagement interventions aimed at enhancing social inclusion through awareness raising (e.g., theatre, radio) [54, 132, 177, 198]. Grey literature, more than research literature, provided evidence of faith communities having a role in social support and inclusion [27, 87, 108].
  Related to stigma and discrimination, *survey respondents* prioritized research topics on how to end discrimination, ways to dismantle public health myths, and how to integrate the voice of PWA to effect social change. *Roundtable* consultation brought into focus that governments and international human rights organizations should use equality and non-discrimination frameworks that exist in all states to promote policies and accountability to such frameworks to secure the human rights of PWA. Expert stakeholders also pointed to the role of academic communities in debunking the myths that perpetuate stigma, and advocacy strategies to disarm harmful myths.
  Although survey items about media and communication as research priorities were not rated highly compared to other items, at the Roundtable participants articulated how media and communication could furtherpromote positive discourses through advocacy, policy and research. According to Roundtable participants, these efforts should: (a) focus on inclusive education and citizenship, (b) cultivate the media as partners, and (c) employ multiple strategies in awareness-raising regarding albinism and human rights.
  Overall, proximal SDOH are dominant in their reported influence on the lived experience of PWA. Bringing variation to the first storyline is the emphasis placed on the more distal (structural) social determinants, such as place and gender equality.

#### II. Distal social determinants of health (SDOH)

The three most commonly cited distal SDOH are place, poverty, and gender equality.

- **The impact of place and rurality.** “Place” is not always included in lists of SDOH, but in the case of albinism, strong evidence exists as to its impact on the realization of the SDOH for PWA. In the *literature* synthesis, 42% (*n* = 90) of sources identified place as mattering in the following ways:
  ◦ **In relation to the prevalence of albinism.** The rates of albinism vary worldwide. In North America and Europe, 1 in every 17,000 to 20,000 people has some form of albinism, and this rate increases in sub-Saharan Africa (as high as 1 in 1000 for selected populations), parts of the Pacific (1 in 700), and among some Indigenous peoples in North and South America (1 in 70 to 1 in 125) [3]. As noted by the UN Independent Expert on Albinism, “an important caveat is that some studies of the frequency of albinism often lack objectivity in their methodology or are incomplete, rendering estimates as best guesses in most instance” (p. 5) [3].
  ◦ **In relation to the amount of sun exposure.** With the predominance of literature on albinism relating to Africa, the concern about sun exposure in tropical climates is frequently mentioned [47, 128, 146] with daily patterns adapted by, for instance, avoiding the outdoors midday. Yet, in places where concern for safety is a constant presence, travelling in the dark brings other risks and worries [92]. Education, as an interrelated SDOH, comes into play when PWA are unaware of their risk for skin cancer and other skin-related conditions. *Amnesty International* reports that in Malawi, most adult people with albinism they interviewed only received information about albinism and preventing skin cancer much later in life when they have already been affected by the harsh African sun [92]. Employment, likewise, relates to place and sun exposure, in places where the main economic activities are subsistence farming and trading at the markets [90]. Limited employment opportunities and vocational training condemn many PWA to outdoor income-generating activities under extended sun exposure [101].
  ◦ **In relation to the explanatory systems at play** (i.e., African ontology). On a broader level, place is related to the explanatory systems that may be at play in relation to causal forces – that cause and are caused by albinism [3, 12]. The literature commonly locates myths and superstitions as characteristic of rural areas where people tend to have less education, and where traditional healers are readily accessed [31, 180]. We take up the matter of explanatory systems in detail in Storyline #2.
  ◦ **In relation to security and protection.** Place—whether rurality or jurisdiction—is also a distal determinant of access to legal services and policing. In Tanzania, the local governance system of street leaders has managed to avert many attacks on the one hand [80], but the state has been criticized for not fully implementing its obligations as a UN member state to prosecute those involved in attacks [61]. The grey literature has been more likely to address security and protection, as to the location of attacks, although the academic has increasing brought attention to these concerns, as well as a call to improve record keeping. Mostert [181], drawing on statistics from *Under the Same Sun*, reports that trafficking of the body parts of PWA have occurred at relatively high levels in Tanzania, Burundi, Kenya, the Democratic Republic of the Congo, Mozambique, Malawi, South Africa, and Swaziland. Isolated reports of killings and attacks have also been documented in Benin, Botswana, Burkina Faso, Cameroon, Egypt, Ghana, Guinea, Ivory Coast, Lesotho, Mali, Namibia, Niger, Nigeria, Rwanda, and Senegal.”
  The *survey* did not elicit place as a primary focus for research, but rural locations tended to be named contextually, accounting for variations in access to vision care, increased risk for skin cancer in tropical climates, government responses, and so forth. Similarly at the *Roundtable*, place and rurality were referenced as context or setting.
- **Poverty as subtext**. As a further variation, bringing some discordance to the storyline of the lived experience of PWA is the impact of *poverty* and deprivation, which are named as an influencing factor to the well-being of PWA in 34% (*n* = 73) of the literature [2, 3, 27, 33, 73, 167, 193]. Four of the 155 *survey* items were specifically related to addressing poverty [Advocacy #9 (Intersectoral advocacy strategies that primarily seek to alleviate poverty), Policy #52 (Develop and implement poverty reduction strategies), Research #109 (Research on how the rapid shift from local, agrarian economies to global market economies relates to the security and wellbeing of PWA), Research #110 (Research on how poverty impacts PWA, and how this can be managed)], In Round 2 of the survey, when experts rated the items, none of the four rated as top priorities, and two of these four items scored in the bottom 2 of their respective clusters (item 9 – advocacy to alleviate poverty), and item 52 – policy for poverty reduction). We interpret this finding of *poverty as subtext* as a systemic issue that stakeholders view as pervasive and difficult to address as a broad goal, but one that can be tackled through more concrete interventions (e.g., ensuring access to health care, education, and employment).
- **Gender equality**. Although the research and grey literature is replete with references to the gendered nature of albinism (e.g., causation blamed on women though both men and women need to carry the genetic mutation; women carrying a disproportionate burden of raising children with albinism, sexual violence), little research through a gender lens has been undertaken since Kromberg et al.’s [211] early work on the response of African mothers to the birth of an infant with albinism. Likewise, little explicit gender analysis re: albinism and human rights (see Ojilere and Saleh [79], as an exception) was located with our search; 8% (*n* = 17) incorporated such an analysis. Gender inequality is also experienced by girls; Franklin et al. [36] report that boys have twice the access to vision support as do girls.
  *Survey* respondents rated “how to support families” as the top priority in the research cluster of Family, and “resilience” as the top priority in the research cluster of psychological wellbeing. (See Table 1). At the *Roundtable*, considerable momentum was built around the imperative of research examining mothering (a project the authors have since begun, see Reimer-Kirkham et al. [212]; Ero et al. [213] as well as in Likumbo et al. [214]).

### Storyline 2 – threats to the security and well-being of PWA through a human rights lens

In the second storyline, concordance exists in relation to the existence of human rights violations (threats of violence are noted in virtually all of the data). This storyline asserts that PWA are human rights claimants, entitled to health, non-discrimination, and full participation in society under multiple human rights instruments (See Table 3). The right to welfare and security is reiterated in the academic and grey literature revealing a consensus regarding the need to address these violations, but also to be aware of the very existence of voices and social forces that would refute this stance. Discordance arises in relation to what the priorities should be in responding to human rights violations. Our analysis reveals that priorities vary according to how the underlying causes of human rights violations are perceived.

**Table 3:** Human rights framing

	**Academic Literature References (*n* = 130)**	**Grey Literature References (*n* = 86)**
**International Instruments**		
Universal Declaration of Human Rights *n* = 41 (19%)	[2, 10, 12, 41, 42, 49, 52, 56, 59, 61, 66, 67, 73, 76, 79, 80, 129, 150, 151, 167, 183–185] *n* = 23 (18%)	[3, 27, 89, 91, 95, 102, 106, 109, 110, 115, 116, 122, 153, 157, 193, 199, 202, 204] *n* = 18 (21%)
Convention of the Rights with Persons with Disabilities *n* = 47 (22%)	[10, 11, 36, 37, 41, 42, 46, 47, 49, 50, 56, 59, 72, 73, 139, 150, 167, 171, 174, 183, 184] *n* = 21 (16%)	[81, 83, 84, 89, 91, 92, 94, 95, 98, 101–103, 106, 108, 109, 113–116, 122, 153, 157, 202, 204, 215, 216] *n* = 26 (30%)
Convention on the Elimination of All Forms of Racial Discrimination *n* = 24 (11%)	[49, 52, 56, 67, 79, 138, 151, 183] *n* = 8 (6%)	[89, 92, 94, 95, 98, 99, 101, 103, 106, 109, 114–116, 153, 157, 204] *n* = 16 (19%)
Convention on Elimination of Discrimination against Women *n* = 20 (9%)	[10, 56, 67, 76, 129, 167, 183] *n* = 7 (5%)	[3, 89, 92, 103, 106, 109, 114–116, 120, 158, 199, 202] *n* = 13 (15%)
Convention on the Rights of the Child *n* = 27 (13%)	[36, 50, 79, 129, 149–151, 183] *n* = 8 (6%)	[3, 81, 89, 91, 92, 95, 99, 101, 103, 106, 108, 109, 114–116, 154, 157, 199, 202] *n* = 19 (22%)
Convention against Torture and other Cruel, Inhuman or Degrading Treatment or Punishment *n* = 13 (6%)	[42, 50, 167, 183] *n* = 4 (3%)	[89, 92, 99, 103, 106, 109, 116, 157, 202] *n* = 9 (10%)
Convention on Civil and Political Rights *n* = 28 (13%)	[31, 42, 49, 52, 56, 67, 73, 79, 129, 151, 183] *n* = 11 (8%)	[3, 89, 91, 92, 95, 96, 99, 106, 109, 115, 116, 157–159, 193, 199, 202] *n* = 17 (20%)
Convention on Economic, Social and Cultural Rights *n* = 21 (10%)	[12, 31, 67, 73, 79, 80, 149, 151] *n* = 8 (6%)	[3, 89, 92, 99, 101, 106, 109, 110, 115, 116, 157, 158, 202] *n* = 13 (15%)
Convention for the Protection of All Persons from Enforced Disappearance *n* = 1 (0%)	*n* = 0 (0%)	[202] *n* = 1 (1%)
Convention relating to the Status of Refuges *n* = 3 (1%)	[183] *n* = 1 (1%)	[94, 122] *n* = 2 (2%)
Convention against Transnational Organized Crime *n* = 2 (1%)	*n* = 0 (0%)	[103, 122] *n* = 2 (2%)
UN Declaration on the Rights of Persons Belonging to National or Ethnic, Religious and Linguistic Minorities *n* = 1 (0%)	[79] *n* = 1 (1%)	*n* = 0 (0%)
**African Instruments**		
African Charter of Human and Peoples’ Rights *n* = 33 (15%)	[31, 45, 49, 50, 52, 59, 61, 67, 73, 79, 127, 139, 149, 150, 169, 178, 184] *n* = 17 (13%)	[84, 88, 92, 95, 98, 101, 115, 118, 119, 124, 153, 186, 191, 195, 202, 217] *n* = 16 (19%)
African Charter on the Rights and Welfare of the Child *n* = 15 (7%)	[11, 36, 48, 80, 149, 151, 183, 184] *n* = 8 (6%)	[92, 99, 115, 124, 163, 193, 202] *n* = 7 (8%)
African Charter on Human and Peoples’ Rights on the Rights of Women *n* = 5 (2%)	[139, 183] *n* = 2 (1%)	[115, 163, 193, 202] *n* = 4 (5%)
African Charter on Human and Peoples’ Rights on the Rights of Persons with Disabilities *n* = 2 (1%)	[59] *n* = 1 (1%)	[119, 185] *n* = 2 (2%)
Regional Action Plan *n* = 20 (9%)	[61, 73, 184] *n* = 3 (2%)	[3, 89, 101, 108, 110, 111, 114, 115, 119–122, 124, 186, 195, 216, 217] *n* = 17 (20%)

The numbering in the table corresponds to the References

#### I. Nature of human rights violations (trauma & violence)

The literature synthesis of academic and grey literatures reveals human rights violations against PWA, on a continuum ranging from stigma to discrimination to social exclusion to violence to murder. Indeed, much of what has already been described in Storyline #1 in relation to the SDOH represents forms of trauma and violence. The literature is focused primarily on violence in regions of Africa, though there is reference to human rights violations elsewhere [115, 118]. The grey literature in particular (e.g., reports from international organizations such as *Amnesty International* and the UN Office of the High Commission on Human Rights) documents the nature of human rights violations against PWA. Physical attacks involve abductions and attempted abductions, grave violations, mutilation, assault, rape, and killings [8, 42, 67, 92, 127, 129, 190, 197, 198]. with reports of escalating violence beginning just over a decade ago [198]. A prominent theme in this literature is that victims, usually children and women, are targeted so that their body parts could be harvested for their allegedly mystical powers and sold to bring good fortune. These attacks are linked to organized crime and witchcraft [3, 39, 61, 67, 120, 197]. Along with the attacks, girls and women are targeted for sexual assaults based on beliefs such as that having sexual intercourse with a PWA could cure AIDS/HIV and other conditions [75, 125, 133, 139, 158, 201]. This violence has been strongly condemned by international and national authorities and is in part the reason for the appointment of the UN Independent Expert on Albinism.

#### II. Priorities vary according to perceived underlying impetuses (causes) of human rights violations

A subtle divergence (discordance) can be understood with the explanatory frameworks by which human rights violations are implied, and in turn, the corresponding priorities to address human rights (See Tables 4 and 5). Dominant in the academic and grey literature is that a context of inadequate attention to the SDOH creates the conditions for violence (70%, *n* = 151), and that interventions such as poverty mitigation, and improved health, social services, education, and employment will protect PWA from such violence, such as attacks. Another common explanation is a lack of knowledge about the genetic cause of albinism (53%, *n* = 114) which leads to the reliance on non-scientific and/or traditional explanations, such as linking the condition to witchcraft and mystical beings like ghosts [56, 77, 141, 185, 190, 217]. Refuting these explanations through public education, genetic counselling and positive media representation are common recommendations, with studies that support this direction. Some authors (32%, *n* = 69) attribute violence to weak governance (with accompanying lack of policing, enforcement, or legal frameworks), with the message that advocacy is needed to hold governments accountable. In this situation, civil society organizations (CSOs) often step in with advocacy efforts, and also with service provision to fill the gaps [206]. Although infrequently cited in the literature, the role of globalization and macro-economic forces (4%, *n* = 8) has also been linked to violence against PWA to expose the mining and fishing industries, and thereby interrupting supply and demand markets that profit from the sale of body parts of PWA (as potions, amulets, or tokens for good fortune [92, 167].

**Table 4:** Storyline 2: explanatory frameworks and corresponding priorities

**Cause (*n* = 216)**	**Academic Literature References (*n* = 130)**	**Grey Literature References (*n* = 86)**	**Survey Items (priorities) (*n* = 155)**
**SDOH** *n* = 151 (70%)	[2, 5–8, 10–12, 29–40, 42–56, 76, 58–62, 64–71, 73–80, 125–146, 148–152, 170, 179] *n* = 85 (65%)	[3, 27, 81–124, 153–164, 187, 190, 193, 197, 198, 201, 206, 216] *n* = 66 (77%)	[9, 11–17, 41–43, 45–48, 50–53, 57–61, 75, 76, 79, 84–87, 89–95, 99, 99, 101–103, 105, 106, 110–116, 143, 153] *n* = 54 (35%)
**Ignorance about albinism** *n* = 114 (53%)	[5, 6, 8, 11, 12, 31–33, 35–37, 39–41, 43–45, 47, 49, 53, 55, 56, 58–62, 64, 66, 67, 69, 73, 74, 77, 78, 125–130, 134, 137–139, 151, 152, 169–172, 174, 178, 181, 185, 218] *n* = 56 (43%)	[3, 27, 81–95, 97–99, 101, 102, 104–110, 114, 116, 118–122, 124, 153, 155–158, 162, 187–189, 191–196, 199, 201–203, 205, 216] *n* = 58 (67%)	[10, 18–27, 36, 49, 56, 78, 81–83, 98, 100, 104, 120, 145–147, 149] *n* = 26 (17%)
**Weak Government** *n* = 69 (32%)	[10, 36, 42–44, 46–51, 56, 60, 61, 66, 67, 73, 74, 76, 78–80, 129, 131, 135, 138, 149–152, 169, 170, 183, 184] *n* = 34 (26%)	[3, 81, 82, 85, 86, 89, 90, 92, 93, 95, 98, 99, 101, 104, 106–111, 114–116, 120–124, 153, 161, 193, 199, 202, 216, 217] *n* = 35 (41%)	[1–6, 8, 28–34, 39, 44, 54, 55, 61–65, 68–74, 97, 107, 137–142, 144, 148, 150–152, 154] *n* = 44 (28%)
**Globalization** *n* = 8 (4%)	[130, 134, 148, 169, 180, 219] *n* = 6 (5%)	[27, 197] *n* = 2 (2%)	[38, 108, 109, 126, 127, 129] *n* = 6 (4%)
**Witchcraft** *n* = 77 (36%)	[1, 2, 11, 12, 31, 33, 36, 37, 39, 42–44, 47, 49, 51, 56, 61, 66–70, 73, 77–80, 128, 129, 131, 136, 139, 141, 146, 148, 167, 169, 171, 173, 174, 180, 183, 209] *n* = 43 (33%)	[3, 27, 89, 90, 96, 98, 99, 101, 104–111, 115, 118–123, 156, 157, 160, 190, 192, 193, 197, 198, 202, 204, 217] *n* = 34 (40%)	[7, 37, 40, 66, 67, 119, 121–125, 130–136] *n* = 18 (12%)
**African Ontology** *n* = 29 (13%)	[10, 31, 33, 38, 47, 52, 56, 66, 67, 71, 77, 80, 126, 128, 131, 140, 148, 152, 173–175, 177] *n* = 22 (17%)	[27, 86, 94, 155, 157–159] *n* = 7 (8%)	[77, 80, 96, 117, 118] *n* = 5 (3%)

The numbering in columns two and three corresponds to the References

The numbering in column four corresponds to Supplementary Materials: Survey Items

**Table 5:** Causal narratives with corresponding priorities

**Causal narratives**	**Corresponding priorities**
Social determinants of health (70%)	• Improved access to health/social services • Better education and employment • Poverty mitigation
Ignorance about albinism (53%)	• Public education • Genetic counselling
Witchcraft (36%)	
Weak governance (32%)	• Advocacy • Civil society organizations fill the service gaps
Globalization (4%)	• Global regulation • International legal frameworks
Witchcraft (36%)	• Law enforcement, collect data re: attacks • Regulation of witchdoctors
African ontology (13%)	• Teaching about the nature of being human • Engaging moral and religious leaders

The most divergence exists about the role of witchcraft and the explanatory frameworks that are employed when referring to witchcraft; there is disagreement in the academic literature on how and how much research should be put into the domain of religion, spirituality, cosmology, and witchcraft. The albinism literature on spirituality and culture is predominantly about the geographic region of Africa, which shapes how spiritual and cultural meanings are viewed, to the extent that they are central to understanding albinism and human rights. The most common term in the literature within the spectrum of spiritual and cultural meanings is “witchcraft”, far exceeding other terms such as religion or religious, spiritual or spirituality, faith, church, pastor, worldview, or ontology. Thirty-six percent (*n* = 77) of the literature referenced witchcraft as the underlying cause of violence, compared to 13% (*n* = 29) that framed violence as stemming from African ontology. Those with closer personal proximity (as PWA themselves, or as scholars from the Global South) to threats to human rights for PWA tend to put much stronger emphasis on the need to attend to this domain.

Round 1 of the *survey* yielded 18 (of 155; 12%) topics that related directly to witchcraft or witchdoctors (emic use of language). In Round 2, respondents scored the items highly in the witchcraft cluster (research clusters scored in the order of stigma, myths, and beliefs; research about advocacy; and research about witchcraft) (see Table 1). North-South incongruence was evident in relation to prioritization for research, in that respondents from the Global South prioritized research on (i) harmful practices and human rights violations and (ii) research on networks, organizations and faith communities that support and advocate for PWA. In comparison, respondents from the Global North prioritized (i) effective advocacy strategies and (ii) research on human trafficking and international law. (See Table 1). With the strong representation of PWA advocates within the survey responses, this discordance is also to be understood as that of proximity to the experience of albinism. *Roundtable* participants confirmed this interpretation, observing that those from the Global South are more likely to be thinking of local, immediate priorities, while those from the Global North may have a broader lens on many countries. Varying causal narratives were expressed at the Roundtable, with specific disagreement as to the relative weighting that should be given to these narratives. All agreed on the need for widespread awareness raising (e.g., through public education, community engagement, media communication), the need to reinforce SDOH, and international and national interventions (state, legal). However, there was divergence on the role of religion, and especially the influence of witchcraft, with PWA and participants from the Global South endorsing more attention to African ontology and witchcraft.

## Discussion: theorizing storylines

The findings of this mixed methods project provide guidance for an intersectoral approach to addressing human rights and albinism, including research priorities, advocacy strategies, and policy initiatives, along two storylines. Teasing out points of concordance and discordance that could make consensus on some of the details allowed us to contribute to a fulsome story of the current albinism and human rights movement. Such an approach accentuates that social change is needed to protect the welfare and security of PWA and that a strong voice for PWA must be included in such social change. Research-advocacy-policy collaboratives, with PWA representation, can accelerate such social change. In the discussion that follows, we theorize deeper understandings of the storylines and offer a way forward in summary.

### Addressing social determinants of health (SDOH)

Evidence on the lived experience of albinism suggests an urgent need to shore up the SDOH, as a route to protecting the human rights of PWA. Key features to be addressed include the proximal determinants of access to vision and skin care, access to education, freedom from stigma and discrimination, and social support and inclusion. Distal determinants of health must also be attended to, in particular the impact of place and rurality, poverty, and gender equality. Compelling evidence now exists that health outcomes are distributed along a social gradient within societies, with those at the bottom (defined by income, occupation, and education) faring most poorly [220]. To enable the largest gain in a society’s health outcomes, as reflected in the 2030 Agenda for Sustainable Development to “leave no one behind,” [221] it is important to prioritize the needs of the most marginalized if societies are to register the largest gain in health outcomes. For PWA, it is not their genetic condition itself that accounts for this social gradient, but rather their *lived experience* of albinism.

The impact of SDOH on PWA needs to be understood at proximal and distal levels and should be conceptualized at scales ranging from the micro (e.g., family support, individual resilience to resist stereotypes and hardship) to the meso (e.g., the availability of health services such as vision and skin screening; education accommodations) to the macro (e.g., rurality, poverty, gender equality). Moreover, meta transnational social and political factors—such as global economics and geopolitical power structures—that affect health and health inequities must be considered, as PWA in parts of sub-Saharan Africa (and elsewhere in low resource countries) are disproportionately and negatively impacted. Here, the spiritual and material come together as some Africans negotiate their socioeconomic survival within the global capitalist system and the dynamics of local power. Separate economic pressures and/or the thirst for power make the myth of body parts of PWA as an ingredient to make potions for upward mobility an attractive pursuit for some [222]. Their body parts offer particularly high instrumental value in this economy of desire [76], thereby increasing their vulnerability to the ultimate denial of human rights – the loss of life.

Scholars [223, 224] are increasingly drawing attention to a need to clarify the “causes of the ‘cause of the cause’ – that is, the processes that historically create and systematically reproduce inequalities [225]. Given the global structures in play, Venkatapuram [220] notes that “health inequalities in a society warrant a scope of moral and ethical concern that goes beyond health care or public health, reaching deep into the basic structures of domestic and, indeed, global society” (p. 269) [220]. A strong grounding in equity and social justice is required to tackle SDOH for PWA as moral motivation and ethical imperative to ensure their wellbeing and security.

Joining a SDOH framework with a human rights approach may operationalize such normative calls to equity and social justice. The World Health Organization’s 2016 report on *Social Justice and Human Rights as a Framework for Addressing Social Determinants of Health* [226] proposes that a wide range of rights can effectively be used to address harmful SDOH; and conversely, addressing SDOH contributes to human rights. A SDOH lens prompts attention to the responsibility of governments to provide sufficient resources and services for their citizens, while a human rights framework has the capacity to hold governments accountable for addressing health inequities. Human rights then become contributors to or “legal determinants” of health (p. 1857) [227]. Our research has clearly noted the intersecting nature of SDOH for PWA and, equally, the intersecting nature of human rights. It is virtually impossible to tease apart, for instance, access to health services, rurality, and poverty. For this reason, a broad range of solutions that threaten the health and human rights of PWA must be put forward as an agenda for research-advocacy-policy work.

### Accounting for explanatory frameworks

The second storyline illustrates the impact of the explanatory frameworks at play. Forsyth and Gibbs [228], in their analysis of sorcery or witchcraft in Papua New Guinea, show how causal narratives “operate to position understandings of events within either the realm of the natural or the realm of the supernatural, and thereby occasion a positioning of the moral dimensions and assignment of blame for the event” (p.2). In the case of albinism in sub-Saharan Africa, causal narratives clustered around two factors: the natural (e.g., ignorance about albinism, weak SDOH, weak government; globalization and macro-economics) and the supernatural (African ontology; beliefs and practices associated with faith traditions). A contribution of our research is to illuminate and diffract these narratives, acknowledging that spiritual/cultural beliefs and practices feature prominently in threats to the human rights of PWA.

African philosopher Imafidon [229], in his exposition of a philosophy of alterity in the case of albinism in Africa, describes the deeply entrenched ideologies regarding humanness, conceptions of the wellbeing of a community, and spiritualized cosmologies as impacting everyday life that have allowed the violent othering of PWA. In African ontology, there is a hierarchy of beings ranging from Supreme Being, to divinities, to ancestors, to manipular forces (spirits), to human persons, to life forms (plants, animals) and, finally, to nonhuman forms, that may appear human but are not fully human (what he refers to as “queer entities”). In this final category are those with disabilities, including those with albinism (see pp. 39). In this framing, if people are not considered fully human, implementing human rights instruments becomes difficult because the enjoyment of such rights is tied to being human. Moreover, when a cosmology of life force theory is at play, explanations for “evil”, harm, or misfortune are not limited to the natural world. Additionally, the cosmology of interrelatedness of entities (human beings, animals, plants, minerals, events, natural and supernatural) results in an interconnected, interlocked African community that may practice exclusion of beings or entities to “protect the socially approved web of relationships from anything that may threaten its harmony and equilibrium” (p.168) [38]. Such a conception of community can mean that because PWA are not considered human, they are not part of a community to begin with, and therefore their social exclusion is justifiable. Imafidon recommends disrupting false ontological representations of albinism that are entrenched in African societies. This disruption must be explored at both individual and societal levels (PWA rising above this false ontological representation and earning worth and respect in their society and society becoming enlightened).

The causal narrative of witchcraft likewise requires nuanced interpretation, situated within this context of spiritualized ontologies. A first matter is that of language and the unsatisfactory capacity of the English language to sufficiently convey the constellation of meanings at play. In this research, for example, we have seen a conflation of references to traditional healers, African traditional religion, and witchcraft. A further tension relates to colonial histories that have taken pejorative, regulating approaches to “witchcraft” [230]. Our research surfaced discordance between the Global North’s and the Global South’s prioritization of witchcraft as focus for research-advocacy-policy. Returning to Forsyth and Gibbs’ [228] observation about natural and supernatural explanatory frames, regardless of truth claims about the existence of the spirit world and any exchange between the natural and supernatural realms, the prescient point is that supernatural explanations are mobilized in ways that result in harmful practices for PWA.

In the cause of protecting PWA, there may be alliances between human rights and faith leaders (whether associated with African Traditional Religion, Islam, or Christianity) that might be mobilized. Spiritual beliefs and practices play a role in the persecution of PWA as well as in the elimination of persecution. Therefore, an approach is needed that asks about harmful practices, and also about practices that are healing, reassuring, or comforting. Specifically, what are the underlying or explicit discursive practices that can be amplified to protect PWA and promote their equity? An intersectoral and intersectional response must be developed to address the root causes of the inequity of PWA; namely, an approach that includes religion. In the words of a survey respondent: “*In Africa, religion is the unseen mover of beliefs, and sometimes stereotypes and discrimination. It is the soft but strong voice that can change situations”.*

## Limitations

Our approach was guided by well-established methods for meta-narrative syntheses [15, 16] and priority-setting surveys [24], but there are several limitations. As a mixed methods project with a large data set, this manuscript cannot present all the rich findings. Although we have paid attention to Global North and Global South interpretations, the reader is reminded that the bulk of the Global South references relate to Sub-Saharan Africa. It is only recently that human rights-related research pertaining to albinism has increasingly emerged from other geographic regions [118]. Because human rights was an inclusion criterion, we have may have missed other studies conducted with other framings. As an example of other framings, Roundtable dialogue amongst researchers extended priorities to include epidemiological research for better data on frequency of albinism, and research on resiliency and quality of life (especially as a trajectory over the years).

## Conclusions

This project resulted in a comprehensive list of recommendations for research, advocacy, and policy (see report) [27]. We conclude with an abbreviated summary of the actions required to address the negative representations, structural inequities, and dangers that PWA face. First, counter-discourses should be developed that critique and undermine the privileging of melano-normativity. The academic community has an important role to play in debunking myths and in translating knowledge. Second, discourses of inclusive difference and systems of inclusive education and citizenship in formal and informal contexts, such as schools and places of worship, should be promoted. This includes cultivating media as partners and agenda setters for alternative positive narratives and engaging moral and political leaders as agents for progressive change. Third, PWA must be made visible and empowered to speak for themselves. This includes their involvement in all spheres of research, advocacy, and policy. Fourth, there is a need to forge local, regional, and global networks of collaboration between researchers, advocates, and policy-makers for integrated, intersectoral, non-zero-sum approaches to the protection of human rights for PWA. Finally, integrating the intersecting frameworks of social determinants of health and human rights will help to foreground their interrelationship as co-requisites for enhancing the lived experiences of PWA.

### Supplementary Information

**Additional file 1.** Supplementary Materials: Matrix.

**Additional file 2.** Supplementary Materials: Survey Items (Round 2).

## Data Availability

All data included in the systematic review are publicly available research findings. The dataset generated by the priority-setting survey is available upon request. The Roundtable Report is available at: A/HRC/40/62/ADD.2.

## Abbreviations

PWA: Persons with albinism
SDOH: Social determinants of health
UN: United Nations
