# Supplementary material for: Mapping a research-advocacy-policy agenda on human rights and albinism: a mixed methods project
Source: Int J Equity Health. 2024 Jan 2;23:1. doi: 10.1186/s12939-023-02064-5 (PMC10762980; doi:10.1186/s12939-023-02064-5)
Supplement: Supplementary file 1 — Additional file 1. Supplementary Materials: Matrix. [file 12939_2023_2064_MOESM1_ESM.docx]

**SUPPLEMENTARY MATERIALS: MATRIX**

| **Reference Number** | **Peer-Reviewed Article** | **Discipline or Field of Study** | **Country of author(s)** | **Geographic region of focus** | **Focus of Article** | **Type of Article** |
| --- | --- | --- | --- | --- | --- | --- |
| 59 | Adelakun OS, Ajayi MAO. Eliminating discrimination and enhancing equality: A case for inclusive basic education rights of children with Albinism in Africa. Niger J Med. 2020;29:2. http://www.njmonline.org/text.asp?2020/29/2/244/287928 | Both Legal | Nigeria | Africa; Nigeria, South Africa as examples | "This paper seeks to establish albinism as a form of disability that should be construed as such within the framework of the Convention on the Rights of Persons with Disabilities" (p. 245). | Discussion |
| 60 | Adenekan TE. Information needs of albinos in the Yoruba ethnic group, Nigeria. International Journal of Technology and Inclusive Education. 2019;8:1. | Philosophy | Nigeria | Nigeria | "This paper explores the perception of Ibule-soro people about albinism and also closely considers the unanswered questions about their rejection" (p. 1385). | Qualitative |
| 165 | Adeyanju B A, Dipo OF, Taiye AM. Health and psychosocial complaints of elderly albinos in Ondo state, Nigeria. Indian Journal of Gerontology. 2015;29:3. | Nursing | Nigeria | Ondo State, Nigeria (Authors note their intention in choosing this area of Nigeria was due to the "numerous cultural orientations and histories of several traditional towns" (p. 370). | "This study examines the health and psycho-social problems that are associated with elderly albinos in Ondo state, the study examines the specific experiences, feelings, coping strategies, cultural influences and attitudes of the society to elderly albinos, as well as medical/nursing benefits that are available for them" (p. 364). | Prevalence |
| 5 | Affram AA, Teye-Kwadjo E, Gyasi-Gyamerah AA. Influence of social stigma on subjective well‐being of persons with albinism in Ghana. Journal of Community & Applied Social Psychology. 2019;29:4. https://doi.org/10.1002/casp.2403 | Psychology | Ghana | Ghana | "The aims of this study were (a) to assess the association between social stigma and subjective well‐being of PWA in Ghana and (b) to explore the mediating role of perceived social support (i.e., family support, friend support, and significant other support) in the association between social stigma and subjective well‐being" (p. 326). | Prevalence |
| 231 | Africa Research Bulletin. Albino killings are spreading to neighbouring states. Blackwell Publishing Ltd, 2009. 46(2), 17880-17881. | Political Science/ Socio-Cultural | Uncertain | Tanzania | Gov't of Tanzania responding to recent murders of PWAs by revoking licenses of Witchdoctors and arresting those accused | Discussion |
| 145 | Arimoro A. Are they not Nigerians? The obligation of the state to end discriminatory practices against persons with disabilities. International Journal of Discrimination and the Law. 2019;19:2. https://doi.org/10.1177/1358229119846764 | Legal | South Africa | Nigeria | "This article aims to, among others, highlight the plight of PWD in Nigeria, the need to protect their rights and guarantee their freedom from discriminatory practices" (p. 89). "This article examines, in the Nigerian context, the treatment of PWD in the country by using various themes. These include their access to education, employment, justice and social amenities. The aim is to bring to the fore the issue of discrimination against PWD in Nigeria and the need for government at the national and subnational levels to implement a legal and policy regime where the rights of PWD would be protected and where their freedom from discrimination is guaranteed" (p. 90). | Discussion |
| 30 | Baker C. Chromatic ambivalence: Colouring the albino. In: Horrocks C. Cultures of colour: Visual, material, textual. New York, Berghahn Books; 2012. p. 143-153. | Literary Studies | UK | Africa | Examines the following authors and their work in terms of their representation of the 'albino body': assine’s Wirriyamu (1976) is set against the background of the final days of colonial Africa, Grainville’s Le Tyran Eternel (1998) and Destremau’s Nègre Blanc (2002) focus on the aftermath of colonial rule, while a second novel by Sassine, Mémoire d’une Peau (1998), presents an uncompromising picture of postcolonial Africa. | Discussion |
| 29 | Baker C. Writing over the illness: The symbolic representation of albinism. In: Twohig P, Kalitzkus V, editors. Social studies of health, illness and disease: Perspectives from the social sciences and humanities. Brill Rodopi; 2008. p. 116-127. | Language studies | UK | Africa | Misrepresentation of PWAs in literature and fixation on the albino body- exploring the ways this occurs and why | Discussion |
| 125 | Baker C. Lund, P. The role of African fiction in educating about albinism and human rights. Journal of Literary & Cultural Disability Studies. 2017;11:3. https://doi.org/10.3828/jlcds.2017.22 | Baker: Language Studies, Lund: Geneticist | UK | Sub-Saharan Africa- Zimbabwe (Takadini); Botswana (Pula) | "Our interest here is in the role that fiction can play in contributing to enhancing understandings of albinism and raising awareness of the human rights of people with albinism in Africa" (p. 272). | Discussion |
| 6 | Baker C, Lund P, Nyathi R, Taylor J. The myths surrounding people with albinism in South Africa and Zimbabwe. Journal of African Cultural Studies. 2010;22:2. https://doi.org/10.1080/13696815.2010.491412 | Various: European Languages and Cultures; Biomolecular and Sports Science; Zimbabwe Albino Association;/ Nursing/ Midwifery | UK & Zimbabwe | South Africa and Zimbabwe | "Examine these myths and trace their impact on the lives of PWA" (p. 169). | Qualitative |
| 166 | Beckham A. Albinism in negro children. The Pedagogical Seminary and Journal of Genetic Psychology. 1946;69:2. https://doi.org/10.1080/08856559.1946.10533389 | Psychology | USA | USA | "To observe the development in a group of albinos during a period of 10 years" (p. 199) | Case Series |
| 31 | Benyah F. Equally able, differently looking: Discrimination and physical violence against persons with albinism in Ghana. Journal for the Study of Religion. 2017;30:161–188. http://dx.doi.org/10.17159/2413-3027/2017/v30n1a7 | Religious Studies | Ghana | Ghana | How inculturation of human rights may help mitigate violence against PWA; addresses both religious beliefs and cultural beliefs | Discussion |
| 126 | Blackenberg N. That rare and random tribe: Albino identity in South Africa. South- North Cultural and Media Studies. 2000;14:2. https://doi.org/10.1080/02560040085310081 | Cultural Studies | South Africa | Africa/South Africa/US | Explores the experience of persons with albinism in terms of identity- touches on racism, colourism, and tensions resulting from skin colour. | Qualitative |
| 146 | Borile S. Exclusion, violence and social utility. The social role of children with albinism in African society. http://www.rivistailluminazioni.it/wp-content/uploads/2020/06/Borile_EXCLUSION-VIOLENCE-AND-SOCIAL-UTILITY-THE-SOCIAL-ROLE-OF-CHILDREN-WITH-ALBINISM-IN-AFRICAN-SOCIETY.pdf (2019). Accessed 01 April 2020. | Linguist, Anthropology, Criminologist | Uncertain | None perse; focus is on "African society" (title) | The focus is on "the sociocultural phenomenon of children with albinism, who bring both bad luck and healing, marks a fate of marginalisation, violence and social exclusion... Some cultures believe their uniqueness gives them healing powers, leading to muti killings (or medicine murder), criminal actions managed by illegal markets and fed by widespread and dominant cultural traditions; their organs are excised for their regenerating, healing and positive powers. Such a culture of violence and ignorance compromises childhood and violates the human, social and civil rights of those who, because of their diversity, are considered elements to be eradicated" (p. 225). | Discussion |
| 127 | Braathen SH, Ingstad B. Albinism in Malawi: Knowledge and beliefs from an African setting. Disability & Society. 2006;21:6. http://dx.doi.org/10.1080/09687590600918081 | Health Research, Living Conditions, and Service Delivery | Norway | Malawi | To examine knowledge, beliefs and behaviour related to people living with albinism in Malawi (p. 599) | Qualitative |
| 7 | Bradbury-Jones C, Ogik P, Betts J, Taylor J, Lund P. Beliefs about people with albinism in Uganda: A qualitative study using the Common-Sense Model. PLoS ONE. 2018;13:10. https://doi.org/10.1371/journal.pone.0205774 | (1) School of Nursing, (2) Head Office, Source of the Nile Union for Persons with Albinism (SNUPA) (3) Advantage Africa, Olney, Buckinghamshire (4) Birmingham Women’s and Children’s Hospital NHS Foundation Trust (5) School of Life Sciences, Albinism in Africa project | (1) United Kingdom, (2) Uganda, (3) United Kingdom (4) United Kingdom (5) United Kingdom | Uganda | "This article reports on the findings of qualitative study conducted in Uganda that addressed this gap in evidence" (p. 2). "Exploring perceptions and beliefs held about the condition formed an important part of the study because it enabled any misconceptions about albinism that lead to discrimination and harmful practices to be challenged" (p. 3). | Qualitative |
| 32 | Brocco G. Albinism, stigma, subjectivity and global-local discourses in Tanzania. Anthropology & Medicine. 2016;23:3. https://doi.org/10.1080/13648470.2016.1184009 | Medical Anthropology | Germany | Tanzania | "This paper aims to show how the subjectivities of people with albinism in Tanzania are shaped and re-shaped through local moral conceptions as well as globalizing (bio)medical explanations of albinism" (p. 229). | Qualitative |
| 128 | Brocco G. Labeling albinism: Language and discourse surrounding people with albinism in Tanzania. Disability & Society. 2015;30:8. https://doi.org/10.1080/09687599.2015.1075869 | Medical Anthropology | Germany | Tanzania | Language and Discourse surrounding PWAs in Tanzania | Qualitative |
| 177 | Brocco G. Notes of despair and consciousness: performativity and visibility of albinism in musical practices. Disability & Society. 2020;35:67-88. https://doi.org/10.1080/09687599.2019.1609422 | Medical Anthropology | Germany | "African countries and other geographical contexts around the world” (p. 70), but with focus on three individuals from Jamaica, Mali and Tanzania | "This article therefore explores the intertwinement of notions of disability identity related to albinism and the enactment of social performativity, visibility and masculinity of the condition in musical productions" (p. 67). "Through an investigation of two musicians with albinism, one from Mali and one from Jamaica, and the life and the performances of a Tanzanian artist with the condition, this article explores the intersectionality of performativity, visibility and masculinity in relation to albinism in various social and geographical contexts. The analysis assumes that albinism is mainly categorized as a disability according to individual accounts collected during previous ethnographic research in Tanzania and other African countries" (p. 69). | Discussion |
| 129 | Bruynell M. The dangers of modern-day belief in the supernatural: International persecution of witches and albinos, Suffolk Transnat'l L. 2012;35:383-420. | Legal | USA | International | Reviews geographic range of victims of occult-based violence (witch and albino), discusses current international efforts and legislation in place, analyzes "present remedies and the dire need for the creation and expansion of international law in this realm" (p. 394). | Discussion |
| 33 | Bryceson DF, Jonsson JB, Sherrington R. Miners' magic: Artisanal mining, the albino fetish and murder in Tanzania. The Journal of Modern African Studies. 2010;48:3. https://doi.org/10.1017/S0022278X10000303 | Bryceson: sociology/  geography; Jonsson: geography and NGO; Sherrington: anthropologist | UK & Denmark | Tanzania | “We discuss the concepts of commodification and fetish creation.  We then interrogate the agency of those involved directly and indirectly in the murders: the miners, some of whom seek albino charms, the waganga, some of whom prescribe and sell the charms, and the albino murder victims" (p. 355). | Discussion |
| 130 | Bucaro S. A black market for magical bones: The current plight of East African albinos. Public Interest Law Reporter. 2010;15:8. http://lawecommons.luc.edu/pilr/vol15/iss2/8 | Attorney | USA | East Africa | Report on the plight of East African PWA | Discussion |
| 61 | Burke J. Persons with albinism: Not ghosts but human beings. In: Izarali, MR, Masakure O, Ibhawoh B, editors. Expanding perspectives on human rights in Africa. 1st ed. Routledge; 2019. p. 158-178. | Social Work | African Australian | Tanzania | This chapter uses analysis of online Tanzanian media reports to capture depictions of people with albinism and the human rights issues that they encounter. | Discussion |
| 131 | Burke J, Kaijage TJ, John-Langba J. Media analysis of albino killings in Tanzania: A social work and human rights perspective. Ethics and Social Welfare. 2014; 8:2. http://dx.doi.org/10.1080/17496535.2014.895398 | Social Work | Australia, South Africa, & Tanzania | Tanzania | "The aim is to explore the involvement of various social actors in violating, or protecting, the right of people with albinism, as well as strategies of preventing and responding to the violence. These explorations are guided by a human rights framework...specific objective is to document and support the endeavors of Tanzanian social workers by making evidence-based recommendations and participate in advocacy towards reducing albino murders in Tanzania" (p. 118). | Discussion |
| 232 | Carnegie CV. The dundus and the nation. Cultural Anthropology. 1996;11:4. http://www.jstor.org/stable/656665 | Anthropology | USA | Jamaica, The Caribbean, and the US | To "explore…the significance of the dundus (and of albinism more broadly) as a symbolic form embedded in a wider social discourse, the geographical and temporal boundaries of which shift as the narrative scrolls between Jamaica, a wider Caribbean, and the United States, as well as between the late 18th century and the present" (p. 472). | Discussion |
| 167 | Chaki FH. Killings of persons with albinism (PWAs) in Tanzania: Deconstructing dominant cause narratives (2007-2015) (Maters Thesis). https://www.semanticscholar.org/paper/Killings-of-persons-with-albinism-(PWAs)-in-cause-Chaki/bdc3ca0f9e9a94df2bac78f48dc80da6ea09e9be (2013). Accessed 24 Jan 2018. | Conflict and peace studies | Tanzania | Tanzania | "To identify dominant narratives around the causes of killings of people with albinism (PWAs) in Tanzania"- focus is from 2007-2015 | Discussion |
| 132 | Chinyowa K, Chivandikwa N. Subverting ableist discourses as an exercise in precarity: A Zimbabwean case study. The Journal of Applied Theatre and Performance. 2017;22:50-61. https://doi.org/10.1080/13569783.2016.1263562 | Chinoyowa: drama and film studies; Chivandikwa: Theatre | South Africa and Zimbabwe | Zimbabwe | "Focuses on ableist discourses and practices seek to 'normalize' the disabled body which is deemed to be 'deviant'" (p. 50). Discusses use of theatre to "subvert ableist discourse" (p. 50). | Discussion |
| 178 | Chisiza Z. Exploring educational theatre & peer learning to combat stigma & myths about albinism in school settings in Malawi. In: Okoye C, Adeyemi S, editors. African Theatre 18. Boydell & Brewer: 2019. p.83-99. | Theatre for Development Practitioner, Lecturer in Drama | Malawi | Malawi | This article implemented "an educational theatrebased and peer-led learning albinism awareness campaign in eleven public schools" (p. 83). | Qualitative |
| 233 | Clark S, Beale J. Albinism and social marginalization. In: Kromberg J, Manga, P. Albinism in Africa: Historical, Geographic, Medical, Genetic and Psychosocial Aspects. Elsevier Inc; 2018. p. 257-270. | Advocates - Standing Voice | UK | Tanzania | albinism and social marginalization- discusses human rights violations explicitly | Discussion |
| 1 | Cohan JA. The problem of witchcraft violence in Africa. Suffolk University Law Review. 2011;44;803-872. https://www.researchgate.net/publication/265076453 | Legal | USA | Africa | Witchcraft violence in Africa | Discussion |
| 8 | Cruz-Inigo AE, Ladizinski B, Sethi A. Albinism in Africa: Stigma, slaughter and awareness campaigns. Special Topics in Tropical Dermatology. 2011;25:79-87. https://doi.org/10.1016/j.det.2010.08.015 | Medicine | USA | sub-Saharan Africa | Aims to draw attention to the dire situation of PWAs in sub-Saharan Africa, heavy focus on access to health care and preventative measures re: skin CA. | Discussion |
| 62 | Dapi LN, Tambe BA, Monebenimp F. Myths surrounding albinism and struggles of persons with albinism to achieve human rights in Yaoundé, Cameroon. Journal of Human Rights and Social Work, 2018;3;11-16. https://doi.org/10.1007/s41134-018-0048-5 | (1,3) Department of Public Health, (2) Centre for Food and Nutritional Research (CRAN), Institute of Medical Research and Medicinal Plant Studies (IMPM), Ministry of Scientific Research and Innovation | Yaoundé, Cameroon | Yaoundé, Cameroon | explore the challenges faced by persons with albinism, with particular reference to their knowledge of albinism, social and health issues and support. | Qualitative |
| 133 | Dart G, Nkanotsang T, Chizwe O, Kowa L. Albinism in Botswana junior secondary schools – a double case study. British Journal of Special Education. 2010;37:2. https://doi.org/10.1111/j.1467-8578.2010.00465.x | Education | UK | Botswana | "This study… considers the educational experience of two particular pupils with albinism in junior secondary schools in Botswana" (p. 77). Considers social, cultural, educational, and some health aspects of pupil experience. | Case Series |
| 2 | Dave-Odigie CP. Albino killings in Tanzania: Implications for security. Peace Studies Journal. 2010;3:68-75. | Peace and Conflict Resolution | Nigeria | Tanzania | "…for the purposes of the paper, albino killing is a topic that falls under the individual (human) aspect of security…. the paper agrees that the notion of security focused on military balances and capabilities need to be broadened to include safety from chronic threats like hunger, disease, repression and protection from sudden and hurtful disruptions in the pattern of daily life" (p. 70). | Discussion |
| 179 | de Groot T, Jacquet W, de Backer F, Peters R, Meurs M. Using visual vignettes to explore sensitive topics: A research note on exploring attitudes towards people with albinism in Tanzania. International Journal of Social Research Methodology. 2020;23:6. https://doi.org/10.1080/13645579.2020.1757250 | Educational Sciences | (1,2,3,5) Belgium, (4) Netherlands | Tanzania | The focus of this article is to apply visual vignette during discussions and interviews. | Qualitative |
| 9 | de Groot T, Jacquet W, Meurs P, Peters, R. Measuring stigma related to people with albinism in Tanzania: A cultural validation study of the EMIC-CSS and SDS among adults. Disability CBR & Inclusive Development. 2020;31:7-29. http://doi.org/10.5463/dcid.v31i1.961 | Educational Sciences | (1,2,3) Belgium, (4) Netherlands | Tanzania | This research aimed at the cultural validation of two Scales to measure stigma related to albinism: The Albinism Social Distance Scale (A-SDS) and the Albinism Explanatory Model Interview Catalogue Community Stigma Scale (A-EMIC-CSS). | Cross-sectional |
| 63 | de Groot T, Meurs P, Jacquet W. The effect of contact interventions on the stigma of people with albinism in Tanzania. Journal of Visual Impairment & Blindness. 2019;113:5. https://doi.org/10.1177/0145482X19874188 | Educational Sciences | Belgium | Tanzania | This article focuses on contact intervention as a strategy to reduce stigma against PWA. | Discussion |
| 147 | de Groot T, Peters R, van Brakel W, Pieter M, Jacquet W. The cultural validation of two scales assessing albinism-related social stigma among high school students in Tanzania. Disability CBR & Inclusive Development. 2019;30:4. http://doi.org/10.5463/dcid.v30i4.861 | (1) Cultural Anthropology and Development Sociology, Educational Science (2). Physiotherapy, (3) Epidemiology (4) Social Agogical Sciences, (5) applied mathematician, educational services | (1,4,5) Belgium, (2,3) Netherlands | Tanzania | "This research aimed to develop a tool to measure albinism-related social stigma among high school students in Tanzania" (p. 11). "This article will specifically focus on the Scale validation for adolescents in secondary school" (p. 12). | Mixed |
| 209 | Dehm S, Millbank J. Witchcraft accusations as gendered persecution in refugee law. Social & Legal Studies. 2018;28:2. https://doi.org/10.1177/0964663917753725 | Law | Australia | Australia, Canada, United Kingdom, United States and New Zealand | "This research examines how claims of witchcraft-related violence were addressed in all available asylum decisions in English, drawn from five jurisdictions. We argue that WRV is a manifestation of gender-related harm; one which exposes major failings in the application of refugee jurisprudence" (p. 202). | Discussion |
| 34 | dos Santos NLP, Moreira MC, Pereira RMM, da Silva ALA. O cuidado à s aúde de pessoas com albinismo: uma dimensão da produção da vida na diferença. Physis - Revista de Saúde Coletiva. 2016;27:2. http://www.redalyc.org/articulo.oa?id=400852156008 | Public Health/  Health Sciences | Brazil | Brazil | discusses health care of people with albinism and family members | Qualitative |
| 64 | Duri F, Makama A. Disabilities and human insecurities: Women and oculocutaneous albinism in post-colonial Zimbabwe. In: Mawere M, Nhemachena A. Rethinking securities in an emergent techno-scientific new world order: Retracing the contours for Africa's hi-jacked futures. 1st ed. Langaa Research & Publishing Common Initiative Group; 2018. p. 77-110. | (1) Anthropology, History (2) uncertain | Zimbabwe | Zimbabwe | "This chapter identifies Zimbabwean women living with albinism as one of the most vulnerable sections of the population. It explores their insecurities in various spheres of life and some of the mitigatory strategies they employ" (p. 77). | Discussion |
| 65 | Estrada-Hernandez N. Psychosocial aspects of physical difference: The experiences of eight adults with albinism in Puerto Rico. Journal of Visual Impairment & Blindness. 2018;112:6. https://doi.org/10.1177/0145482X1811200605 | Counselling, Rehabilitation, and student development | USA | Puerto Rico | "This article explored the experiences of eight persons with albinism in Puerto Rico in their quest for educational, social, and employment opportunities" (p. 701). | Qualitative |
| 168 | Estrada-Hernandez N, Harper DC. Research on psychological and personal aspects of albinism: A critical review. Rehabilitation Psychology. 2007;52:3. https://doi.org/10.1037/0090-5550.52.3.263 | Counselling, Rehabilitation, and student development | USA | Global | "...a critical review of the research literature, examining factors associated with adaptation to the albinism condition" (p. 263). | Systematic Review |
| 35 | Ezeilo BN. Psychological aspects of albinism: An exploratory study with Nigerian (IGBO) albino subjects. Social Science and Medicine. 1989;29:9. https://doi.org/10.1016/0277-9536(89)90026-9 | Social Sciences | Nigeria | Nigeria | "Examines the phenomenon of albinism from the albino's viewpoint" (p. 1129) | Qualitative |
| 169 | Fayoyin A, Ihebuzor N. Advocacy for minorities in Africa: Issues and lessons in advancing the rights of albinos in Tanzania and Osus in Nigeria. Asia Pacific Journal of Research. 2014;1:17. | Fayoyin: Advocacy & social development, strategic communication, and impact of new media on oramedia; Ihebuzor: Education and development specialist, public policy analysis | Southern Africa and Tanzania/Nigeria | Tanzania and Nigeria | Explores various advocacy strategies used to "influence public discourse and broader social change towards two minority groups: Albinos in Tanzania and Osus in Nigeria" (p. 113). | Systematic Review |
| 148 | Fioravanti S. The long walk to inclusion of people with disabilities in Kenya, with a particular emphasis on children's right to education (Master's thesis). https://thesis.unipd.it/handle/20.500.12608/25200?1/SERENA_FIORAVANTI_2016.pdf (2016). Accessed 24 Jan 2018. | Modern Languages ​​for International Communication and Cooperation | Italy | Kenya | It "aims to bring to light the condition of people with disabilities in Kenya" (p. 1). | Qualitative |
| 36 | Franklin A, Lund P, Bradbury-Jones C, Taylor J. Children with albinism in African regions: Their rights to ‘being’ and ‘doing’. BMC International Health and Human Rights. 2018;18:2. https://doi.org/10.1186/s12914-018-0144-8 | Franklin and Lund: Health and Life Science; Bradbury-Jone and Taylor: Institute of clinical sciences | England | African Regions | Focus on Children and young people with albinism- using a "social model of disability with discussion on human rights to address the lacuna surrounding the psychosocial and daily experiences of [PWA]" (p. 1). | Discussion |
| 37 | Greenwood M, Fakih B, Steff M, Bechange S, Mrisho, M. Hear my voice: A community-based participatory study gathering the lived experiences of people with disabilities and older people in Tanzania. Knowledge Management for Development Journal. 2016; 11:2. | Greenwood: Education/ Social Inclusion; Fakih: Social Sciences/Public Health; Steff: Education Psychology; Bechange: Health and developmnt programme specialist; Mrisho: Epidemiologist | UK, Tanzania, Southern Africa | Tanzania | What is the "lived experiences of people with disabilities and older people in Tanzania, in this case as captured and expressed by them" (p. 64)? | Qualitative |
| 234 | Gruenbaum O. Commonwealth update. The Commonwealth Journal of International Affairs. 2018;107:4. https://doi.org/10.1080/00358533.2018.1501859 | editor of the Commonwealth Update and a member of the editorial board of The Round Table | UK | International | The focus of this article is to provide a commonwealth update internationally of reported attacks and deaths, with a section for PWA. | Discussion |
| 38 | Imafidon E. Dealing with the other between the ethical and the moral: Albinism on the African continent. Theoretical Medicine and Bioethics. 2017;38:2. https://doi.org/10.1007/s11017-017-9403-2 | Philosophy, Ethics | Nigeria | Africa | "To explore how the uneasiness and consequent indifference about the other play out on the African continent with a particular attention to albinism" (p. 164) | Discussion |
| 235 | Imperato PJ, Imperato GH. Twins, hermaphrodites, and androgynous albino deity: Twins and sculpted twin figures among the Bamana and Maninka of Mali. African Arts. 2008;41;40-49. | Malian history, society, and art | US | Mali | Ancient deities-- history of twins, hermaphrodites, and albinos among the Bamana and Mannka of Mali | Discussion |
| 66 | Kaigoma DD. Violence against people with albinism in Tanzania: A model to combat dehumanization (Master's thesis). https://www.proquest.com/openview/dda5518030c042a10023af873435ad3a/1?pq-origsite=gscholar&cbl=18750&diss=y (2018). Accessed 22 March 2020. | Master of Arts | Alaska | Tanzania | "This paper aims to educate the local culture on the nature of albinism, to encourage respect for human dignity, and to invite the Catholic Church to be an active voice speaking for people living with albinism" (p. 4). | Discussion |
| 149 | Kajiru I, Mubangizi JC. Human rights violations of persons with albinism in Tanzania: The case of children in temporary holding shelters. African Human Rights Law Journal. 2019;19:246-266. http://dx.doi.org/10.17159/1996-2096/2019/v19n1a12 | (1) Applied Human Science (2) Law | South Africa | Tanzania | "This article explores the challenges faced by children at these temporary holding shelters and the extent to which their rights are violated" (p. 246). | Discussion |
| 67 | Kajiru I, Nyimbi I. The impact of myths, superstition and harmful cultural beliefs against albinism in Tanzania: A human rights perspective. Potchefstroom Electronic Law Journal. 2020;23:1-27. http://dx.doi.org/10.17159/17273781/2020/v23i0a8793 | Law | Tanzania | Tanzania | "This article discusses the nature of superstition and myths about PWA in Tanzania and how some societies have perpetrated discriminatory acts against PWAs as an outcome of these false beliefs. The paper discusses the ramifications of these in the context of the rights of PWA. The paper further explains the conflict between superstitions, harmful cultural beliefs and human rights" (p. 3). | Discussion |
| 68 | Kiishweko RR. Albinism in Tanzanian higher education: A case study (Doctoral dissertation). http://catalog.ihsn.org/citations/87398 (2016). Accessed 22 March 2020. | Education and Social Work | England | Tanzania | "This research attempts to contribute to existing literature and construct new insights into albinism and higher education" (p. ii). | Qualitative |
| 39 | Kisanga P, Mbonile MJ. Impact of interventions of the murder of people with albinism in Lake Victoria: A case of Shinyanga region. Journal of the Geographical Association of Tanzania. 2017;37:56-73. | Education and Geography | Tanzania | Tanzania (specifically Lake Victoria, Tanzania in Shinyanga Region and Kaham Districts) | To investigate "the impact of interventions of the murder of PWAs in Lake Victoria particularly Shinyanga region" (p. 57) | Qualitative |
| 236 | Kromberg J. Albinism in the South African negro: IV. Attitudes and the death myth. Birth Defects: Original Article Series. 1992;28:159-166. http://dx.doi.org/10.1017/S0021932000014838 | Human Genetics | South Africa | South Africa | examine the attitudes of South African youth towards albino youths as well as examine the attitudes of young albinos towards themselves | Prevalence |
| 40 | Kromberg J. Albino youth in Soweto: Some features of their adjustment (Master’s thesis). https://citeseerx.ist.psu.edu/viewdoc/download?doi=10.1.1.973.4536&rep=rep1&type=pdf (1977). Accessed 24 Jan 2018. | Social Work/  Human Genetics | South Africa | South Africa (Soweto) | “The purpose of the present study was to investigate some features of the adjustment of the young albino in Soweto, and to determine whether he differed significantly in any way compared to normally pigmented young people” (p. iii). | Mixed |
| 170 | Kromberg J. Interventions: Preventive, management, empowerment, advocacy and support services. In: Kromberg J, Manga P. Albinism in Africa: Historical, Geographic, Medical, Genetic and Psychosocial Aspects. Elsevier Inc; 2018. p. 271-293. | Human Genetics | South Africa | Africa/ International | Current intervention strategies to address needs of PWA. | Discussion |
| 41 | Kromberg J. Psychosocial and cultural aspects of albinism. In: Kromberg J, Manga P. Albinism in Africa: Historical, Geographic, Medical, Genetic and Psychosocial Aspects. Elsevier Inc; 2018. p. 171-201. | Human Genetics/  Social Work` | South Africa | Africa | Psychosocial and cultural aspects of albinism | Discussion |
| 237 | Kromberg J, Manga P. Summary and conclusion. In: Kromberg J, Manga P. Albinism in Africa: Historical, Geographic, Medical, Genetic and Psychosocial Aspects. Elsevier Inc; 2018. p. 308-325. Elsevier Inc; 2018. p. 309-325. | Human Genetics | South Africa | Africa/ International | This is the last chapter of the book and provides a summary. They also discuss “potential gaps in our knowledge and propose possible future research, ranging across the psychosocial, medical, biological, and molecular fields. Finally, possible future developments in the field are covered” (p. 310). | Discussion |
| 211 | Kromberg J, Zwane EM, Jenkins T. The response of black mothers to the birth of an albino infant. American Journal of Diseases of Children. 1987;141:8. | Human Genetics | South Africa | South Africa | Response of "black mothers" to their albino infants over 15 months. | Mixed |
| 42 | Larson S. Magic, mutilation and murder: A case for granting asylum to Tanzanian Nationals with albinism. Pace International Law Review Online Companion. 2011;3:1-29. https://core.ac.uk/download/pdf/46710291.pdf | Law | US | Tanzania | Argues that (1) the "cruel, inhuman, degrading, and discriminatory treatment of Tanzanian nationals with albinism constitutes persecution under American asylum law" and that (2) such PWAs should "be entitled to eligibility for asylum in the US" (p. 1). | Discussion |
| 43 | Lynch P, Lund P, Massah B. Identifying strategies to enhance the educational inclusion of visually impaired children with albinism in Malawi. International Journal of Educational Development. 2014;39:226-234. https://doi.org/10.1016/j.ijedudev.2014.07.002 | Lynch: Education; Lund: Epidemiology; Massah: Albino Association of Malawi | UK and Malawi | Malawi | Educational Inclusion of children: ID barriers to accessing education and determine strategies to overcome these barriers | Qualitative |
| 134 | Machoko CM. Albinism: A life of ambiguity – a Zimbabwean experience. African Identities. 2013;11:3. http://dx.doi.org/10.1080/14725843.2013.838896 | Religious Studies | Canada | Zimbabwe | People with albinism live in ambiguity: on the one hand, while PWA were sometimes seen in a positive spiritual/cultural light, they were also raped and murdered for personal gain, and killed because they were seen as a curse. This was all because "albinism was linked to water spirits and ascribed celibacy" (p. 318). | Qualitative |
| 238 | Maja-Pearce A. Disabled Africa: Rights not welfare. Index on Censorship. 1998; 27:177-195. | Journalism | Nigeria | Various countries in Africa | To explore the problem of how various disabilities -- such as blindness, deafness, albinism, and even being a woman -- are handled in Africa, including what various organizations and initiatives have achieved. | Discussion |
| 180 | Mariki MS. The tragedy of elder abuse and witchcraft accusations: A challenge to church's mission in the Elct, Elvd Sukumaland, Tanzania (Master's thesis). https://mfopen.mf.no/mf-xmlui/bitstream/handle/11250/2447232/AVH5010-1105-Mariki-navn.pdf?sequence=1 (2017). Accessed 24 Jan 2018. | Theology | uncertain | Sukumaland, Tanzania | "This study aims at investigating on the church’s mission work as challenged by the tragedy of witchcraft and mistreatment of the elderly people in Sukumaland, seeking on what and how to meet the challenges, the approaches and implementations of the planned strategies by the church to encounter the problem" (p. 6). | Qualitative |
| 69 | Masanja MM, Imori MM, Kaudunde IJ. Factors associated with negative attitudes towards albinism andeople with albinism: A case of households living with persons with albinism in Lake Zone, Tanzania. Open Journal of Social Sciences. 2020;8:4. https://doi.org/10.4236/jss.2020.84038 | Governmnt | Tanzania | Tanzania | It "aimed at assessing attitudes towards albinism and people living with albinism and how different factors are associated with attitudes towards albinism and people with albinism among households’ members in households living with persons with albinism" (p. 524). | Cross-sectional |
| 70 | Masanja MM, Imori MM, Kaudunde IJ. Lifelong agony among people with albinism (PWA): Tales from Lake Zone in Tanzania. Journal of Social and Political Sciences. 2020;3:2. | Governmnt | Tanzania | Tanzania | "This paper presents explanations and experiences from People with Albinism (PWA)" (p. 329). | Qualitative |
| 150 | Mashegede C. Towards the creation of socially inclusive communities: A study focusing on social inclusion of children with albinism in Tanzania (Master's thesis). http://www.diva-portal.org/smash/record.jsf?pid=diva2%3A1464575&dswid=4746 (2020). Accessed 22 Mar 2020. | Peace and Development Work | Sweden | Tanzania | "A rights-based conceptual framework for the social inclusion of children helped to identify challenges faced by children with albinism in Tanzania as well as to advance a new understanding of the voices and knowledge emerging from the Global South" (p. 3). | Qualitative |
| 218 | Mazibuko NG, Kromberg J. A personal perspective: Living with albinism. In: Kromberg J, Manga P. Albinism in Africa: Historical, Geographic, Medical, Genetic and Psychosocial Aspects. Elsevier Inc; 2018. p. 295-305. | (1) NGO Representative (2) Human Genetics | South Africa | Africa/South Africa | discusses Nomasanto Mazibuko's personal experience- her past and current advocacy efforts as a PWA | Discussion |
| 44 | Mesaki S. Witchcraft and witch-killings in Tanzania (Doctoral dissertation). https://books.google.ca/books/about/Witchcraft_and_Witch_killings_in_Tanzani.html?id=zbg0cgAACAAJ&redir_esc=y (1993). Accessed 24 Jan 2018. | Anthropology | Tanzania | Tanzania | "This is a dissertation about the origins, historical developments and current status of belief and practices of witchcraft and related phenomenon in Tanzania. The main argument is that witchcraft is a socially constructed reality which baffles many of the ruling elites in Africa" (p. 1). | Qualitative |
| 71 | Miller NP. “Other” white storytellers: Emancipating albinism identity through personal narratives. Communication Quarterly. 2019;67:2. https://doi.org/10.1080/01463373.2018.1533486 | Communication Studies | USA | none perse but the two individuals analysed were from "African descent" (p. 126) | "This study analyzes the emancipatory rhetoric within TEDx talks presented by albinic, Black fashion models, Diandra Forrest and Shaun Ross" (p. 123). | Discussion |
| 45 | Miller NP. Un-othering the albino: How popular communication constructs albinism identity [Doctoral dissertation). http://aquila.usm.edu/dissertations/1372 (2017). Accessed 24 Jan 2018. | Communication Studies | U.S. | None per se; media discussed prouced in the U.S. | Essentially, "whiteness operates rhetorically and ideologically in popular discourse to create negative verbal and visual trope of albinism, called the albino trope" (p. ii). | Discussion |
| 46 | Morley L, Croft A. Agency and advocacy: Disabled students in higher education in Ghana and Tanzania. Research in Comparative and International Education. 2011;6:383-399. http://dx.doi.org/10.2304/rcie.2011.6.4.383 | Education and Social Work | United Kingdom | Ghana and Tanzania | “In research, links between higher education access, equalities and disability are being explored by scholars of the sociology of higher education… This article is based on a review of the global literature on disability in higher education and interview findings from the project ‘Widening Participation in Higher Education in Ghana and Tanzania: developing an Equity Scorecard’, funded by the Economic and Social Research Council and the Department for International Development” (p. 383). | Discussion |
| 181 | Mostert. MP. The plight of Africans with albinism. Human Life Review. 2019;45:2. | Special Education | USA | Africa | examining the issues affecting persons with albinism, with the focus on the attacks and trafficking. | Discussion |
| 171 | Mostert MP, Weich MM. Albinism in Africa: A proposed conceptual framework to understand and effectively address a continental crisis, African Disability Rights Yearbook. 2017;5:101-117. http://doi.org/10.29053/2413-7138/2017/v5nla6 | Disability | U.S. (Mostert); South Africa (Weich) | Africa (several; Malawi, Kenya, Burundi, and Tanzania used as examples) | "... we propose an initial conceptual framework that unpacks crucial connections among related variables impacting trafficking in albino body parts in Africa, and then use the framework to suggest areas of emphasis to reduce and eliminate the trafficking of these body parts in Africa" (p. 101). | Discussion |
| 72 | Mswela M. Does albinism fit within the legal definition of disability in the employment context? A comparative analysis of the judicial interpretation of disability under the SA and the US non-discrimination laws. Potchefstroom Electronic Law Journal. 2018:21:1-37. http://dx.doi.org/10.17159/17273781/2018/v21i0a3222 | Law | South Africa | "Reviews the nature of disability claims in the workplace on grounds of albinism in the United States context" (p. 1). | "The objective of such an analysis is to understand the construction of disability under the Employment Equity Act in order to shed light on whether people with albinism qualify for the protection, which is afforded to people with disabilities in the work place" (p. 1). "The article also explores the mandate of the Convention on the Rights of Persons with Disabilities in as far as it relates to the social construction of disability" (p. 4). | Discussion |
| 182 | Mswela M. Tagging and tracking of persons with albinism: A reflection of some critical human rights and ethical issues arising from the use of the global positioning system (GPS) as part of a solution to cracking down on violent crimes against persons with albinism. Potchefstroom Electronic Law Journal. 2019;22:1-27. http://dx.doi.org/10.17159/17273781/2019/v22i0a5206 | Law | South Africa | South Africa | "In South Africa, this article proposes the extension of the application of the use of the Global Positioning Tracking System to persons with albinism as a strategy to halt the continued attacks against this population" (p. 1). The author looked into the ethical issues relating to GPS use to track PWA with the emphasis to rights relating to the right to privacy, liberty, equality and dignity (p. 1). | Discussion |
| 172 | Mswela M. The ‘evil albino’ stereotype: An impediment of the right to equality. Medicine and Law. 2013;32:79-93. | Law | Uncertain. | None per se. | "This article address the 'evil albino' plot device or albino bias as portrayed in films and explores how such labelling stimulates societal intolerance and discrimination against People Living With Albinism" (p. 79). | Discussion |
| 239 | Mtetwa E, Ruparanganda W. Disability and the quest for bureaucratic representation in Zimbabwe. The Indian Journal of Social Work. 2016;77:39-52. | Social Work and Sociology | Zimbabwe | Zimbabwe | "It is the intention of this paper to articulate the central role and function of self-representation in the bureaucracy as an avenue through which the marginalised can gain entry into the mainstream of social life" (p. 40). | Qualitative |
| 73 | Mubangizi JC, Kajiru I. Human rights education and the plight of vulnerable groups with specific reference to people with albinism in Tanzania. International Journal of Discrimination and the Law. 2020;20:2-3. https://doi.org/10.1177/1358229120948691 | Law | South Africa | Tanzania | "This paper explores the nature and extent of discrimination and human rights violations of people with albinism in Tanzania in the context of the relevant legal framework available for their protection. Using people with albinism in Tanzania as a proxy, the paper argues that there is a need for human rights education not only to empower vulnerable people to defend and protect their rights but also to sensitize societies to respect and not violate the rights of such people" (p. 137). | Discussion |
| 173 | Mucina DD. Albinism: An erasable childhood. The Journal of Pan African Studies. 2015;8:3. | Child and Youth Study | Canada | Africa | "I have no formulated answers but I want us to make the childhood of children with albinism matter to us. I want us to question the social political interactions and interpretations that allow us to voice violence, which lead us to murder children with albinism, while we who profess to be Ubuntu remain silent. In our silence, we are complicit in the erasure of children with albinism.... Instead, let us dialogue about the problem and the actions we can take collectively together to address this problem of marking our children for death" (p. 276). | Discussion |
| 47 | Mulemi BA, Ndolo UM. Albinism, witchcraft, and superstition in East Africa: Exploration of bio-cultural exclusion and livelihood vulnerability. https: www.academia.edu/7332091/albinism_witchcraft_&superstition_East_Africa (2014). Accessed 24 Jan 2018. | Arts and Social Sciences | Kenya? | Mwanza and Mtwara regions in Tanzania | "The main purpose of the ethnography was to explore the patterns of beliefs and  superstitions that affect the livelihood and quality of life of persons with albinism and their families in East Africa. Theories of stigma (Goffman 1963, 1997), social and bio-cultural exclusion are used in the ethnography sought to explore the adversities that people with albinism encountered and how they attempted to cope with them" (p. 13). | Qualitative |
| 135 | Mwaipopo RN Lihamba A, Njewele DC. Equity and equality in access to higher education: The experiences of students with disabilities in Tanzania. Research in Comparative and International Education. 2011;6:415-629. http://dx.doi.org/10.2304/rcie.2011.6.4.415 | Mwaipopo: Sociology & Anthropology, Lihamba: Public Services, Njewele: Fine and Performing Arts, Social Sciences | Tanzania | Tanzania & Ghana | "This article analyses the process of access into higher education institutions and outcomes in terms of representation in higher education institutions by students with disabilities. In doing so, it seeks to explore the meaning and outcomes of policies related to higher education institutions in Tanzania in terms of their stated equality ideals and achievements in practice" (p. 415) | Mixed |
| 136 | Myrhe KC. The power of a severed arm: Life, witchcraft, and Christianity in Kilimanjaro. In: Rio K, MacCarthy M, Blanes R. Pentecostalism and witchcraft: Spiritual warfare in Africa and Melanesia. Palgrave Macmillan; 2017. p.163-188. | Anthropology of Religion | Norway | Africa- Tanzania | Witchcraft, Christianity, Catholicism-- challenges notion of "occult economy"; "The circulation of body parts is obviously acute in the albino murders, which raises the question of whether they primarily concern the form and being of persons, rather than matters of economics and forms of representation" (p. 167-168). | Discussion |
| 74 | Nakkazi E. People with albinism in Africa: Contending with skin cancer. World Report. 2019;314:10198. https://doi.org/10.1016/S0140-6736(19)31941-5 | Literary | Uncertain | Africa | This article provides information about cancer and its prevalence with PWA. They also provide information of the work of UTSS. | Discussion |
| 137 | New Internationalist. Tanzania: Rights of albinos. https://newint.org/columns/currents/2008/08/01/tanzania (2008). Accessed 24 Jan 2018. | Uncertain (no author is given) | Uncertain | Tanzania | To explore the issue of discrimination against PWA in Tanzania. | Discussion |
| 48 | Ng’ondi NB. Child protection in Tanzania: A dream or nightmare. Children and Youth Services Review. 2015;55:10-17. http://dx.doi.org/10.1016/j.childyouth.2015.05.012 | Social Work | Tanzania | Tanzania | "This study examined how effective child protection measures have been in alleviating all forms of violations against children" (p. 10) in Tanzania. | Prevalence |
| 75 | Nichols-Belo A. “Witchdoctors” in white coats: Politics and healing knowledge in Tanzania. Medical Anthropology. 2018;37:8. https://doi.org/10.1080/01459740.2018.1476974 | Anthropology | USA | Tanzania | The author "describes how healers locate their knowledge in experience, ancestors, and spirits, while the state imagines a future where traditional healers are formally educated and practice in white uniforms" (p. 1). | Discussion |
| 184 | Nkrumah B. Anywhere but here: A calculus for protection of children with albinism. Journal of International Migration and Integration. 2020. https://doi.org/10.1007/s12134-020-00773-2 | Political Studies | South Africa | none perse but mentions "third countries" (p. 2) and Africa context | "The paper argues that the UN Refugee Agency must partner with western countries in resettling these children and similarly situated persons. The focus on CwA is informed by their limited physical strength to ward off their attackers, thereby making them more vulnerable to abduction and related atrocities" (p. 1). | Discussion |
| 151 | Nkrumah B. How did we get here? Reflections on the UDHR and South Africans with albinism. South African Journal of Criminal Justice. 2019;32:2. | Political Studies | South Africa | South Africa | "Assess whether the UDHR, after seven decades, is still relevant today in safeguarding the rights of SAwA at the national level" (p. 184). | Discussion |
| 76 | Nkrumah B. ‘Hunted like animals’: The conundrums of countering crimes against albinistic persons in the era of the UDHR. International Journal of Law, Crime and Justice. 2018;55:52–59. https://doi.org/10.1016/j.ijlcj.2018.09.002 | Political Studies | South Africa | none perse but focused on Africa | The paper assesses some of the conundrums of the application of the UDHR and "makes recommendation on how to overcome them in order to forestall this crime" (p. 52). | Discussion |
| 183 | Nkrumah B. The hunted: UDHR and Africans with albinism. International Migration. 2018b;57:192-212. https://doi.org/10.1111/imig.12521 | Political Studies | South Africa | Africa | "The article argues that, although African governments remain the primary custodians of Africans with human albinism, the burden of violence often visited on this group constitutes “persecution” under the UDHR and therefore the international community has a role to play in safeguarding this group" (p. 192). | Discussion |
| 50 | Ntinda RN. A critical analysis for the effective implementation of the international convention on the rights of persons with disabilities (Master’s dissertation). 2013. Accessed 24 Jan 2018. | Law | Namibia | Namibia | "The study attempts to critically analyse the current disability legal framework to determine whether it is sufficient and compatible for the effective and efficient implementation of the ICRPD to ensure the reasonable accommodation and protection of persons with disabilities in Namibia or is it lacking both in substance, implementation or application. Based on this, the study further attempts to determine as to whether or not there is a need for review and reform of the disability legal framework in order to about such effective and efficient implementation of the ICRPD" (p. 5). | Qualitative |
| 138 | Ntinda RN. Customary practices and children with albinism in Namibia: A constitutional challenge? https://citeseerx.ist.psu.edu/viewdoc/download?doi=10.1.1.616.6635&rep=rep1&type=pdf (2009). Accessed 24 Jan 2018. | Law | Namibia | Namibia | "This paper will focus on various cultural practices and superstitions surrounding children with albinism in Namibia, which stigmatise and discriminate against them on a daily basis. The intention of this article is to find an answer to whether the customary practices in question present a constitutional challenge" (p. 243). | Discussion |
| 49 | Ntinda RN. The rights of people with albinism: A conceptual and rights based comparative analysis (Bachelor’s dissertation). https://www.academia.edu/33686673/THE_RIGHTS_OF_PEOPLE_WITH_ALBINISM_A_CONCEPTUAL_AND_RIGHTS_BASED_COMPARATIVE_ANALYSIS (2011). Accessed 24 Jan 2018. | Law | Namibia | Namibia | "This dissertation will therefore investigate as to whether the rights of people with albinism are truly protected. Should people with albinism be regarded as vulnerable people and should they be classified as people with a disability" (p. VII)? | Qualitative |
| 77 | Nyakundi KD. Portrayal of disability (albinism) in Goro Wa Kamau's ghost and the fortune hunters (Master thesis). http://erepository.uonbi.ac.ke/handle/11295/109452 (2019). Accessed 22 Mar 2020. | Arts and Literature | Kenya (University of Nairobi) | None perse; it’s a book review | "The study investigates the relationship between portrayal of disability in literature and the perception or image created towards characters with disability… This research focuses on the impact of negative representation and more especially, on ways of creating new interpretations of disabled characters in literature. In this research therefore, we examine the role of the disabled child character in deconstructing disability in Goro Wa Kamau’s novel Ghost and the fortune Hunters. we explore the strategies employed by the disabled character to deconstruct existing stereotypes and misrepresentations in understanding albinism in the text" (p. vi). | Discussion |
| 78 | Nyamu IK. Competing intergenerational perspectives of living with albinism in Kenya and their implications for children’s lives. Childhood. 2020;27:4. https://doi.org/10.1177/0907568220931580 | Development Studies | South Africa | Kenya | "Using generationing as a theoretical lens, this article explores how generational relationships mediate children’s experiences of living with albinism in the context of harmful cultural practices, disability politics and adult-defined activism. Three social institutions which structure generational interactions – the family, the school and the state – are analysed" (p. 435). | Qualitative |
| 51 | Oestigaard T. Witchcraft, witch killings and Christianity: The works of religion and parallel cosmologies in Tanzania. In: Stahl, M. Looking Back, Looking Ahead- Land, Agriculture and Society in East Africa. The Nordic-Africa Institute; 2015. p. 182-199. | Rural and Agrarian Change; archaeology | Sweden? (Nordic Africa Institute) | Tanzania | "As I will argue, Christianity and the world of the ancestors, including the practice of witchcraft, operate on different premises and affect this world in substantially different ways. Precisely because of this, they can coexist and work hand in hand" (p. 184). | Discussion |
| 79 | Ojilere A, Saleh MM. Violation of dignity and life: Challenges and prospects for women and girls with albinism in Sub-Saharan Africa. Journal of Human Rights and Social Work. 2019;4:5. https://doi.org/10.1007/s41134-018-0085-0 | Law | Nigeria | Sub-Saharan Africa | "This paper explores common myth-based violations against women with albinism in Africa. It argues that these women suffer the tragedy of double prejudice and special violations to dignity and life, contrary to standard templates of international human rights" (p. 147). | Discussion |
| 52 | Olagunju A. ‘Being different’: realities of life experiences as constructed by persons with albinism in Nigeria (Doctoral dissertation). https://chesterrep.openrepository.com/handle/10034/623072 (2019). Accessed 22 Mar 2020. | Religion (inferred) | Nigeria (inferred) | Yoruba people of Southwestern Nigeria | "This paper is a Biblical response to the myth and discrimination against the Albinos among the Yoruba people. The paper involves interview of some Albinos to ascertain the kind of abuses they suffer. It also makes use of on-line materials such as e-book, newspapers and articles that focus on human right of the Albinos in Africa, and more especially among the Yoruba people of Southwestern Nigeria. This paper recommends that the Albinos have right to life and they should be treated equally like any nonpigmented persons wherever they found themselves" (p. 46). | Discussion |
| 152 | Olagunju OS. Towards a biblical response to myth and discrimination against the human right of albinos in Yorubaland. Journal of Studies in Social Sciences. 2012;1:46-58. | Philosophy | uncertain | Nigeria | "This research aimed to understand how the realities of being a PWA in Nigeria could be conceptualised based on their life experiences to develop a substantive theory of their social wellbeing status" (p. 10). | Qualitative |
| 208 | Olaiya K. Commodifying the “sacred.” Beatifying the “abnormal”: Nollywood and the representation of disability. The Global South. 2013;7:137-156. http://www.jstor.org/stable/10.2979/globalsouth.7.1.13 | Drama | Uncertain (no professional information given for author) | Nigeria | "This paper examines the representations of disabilities and the disabled in the Nigerian film industry" (p. 137). "I argue that disability in Nigeria is a “socio-cultural and as well as a physical” problem and contend that its representations in Nollywood exploit and simultaneously beatify disabled actors. While Nollywood perpetuates some myths about disability and the disabled, it economically empowers disabled actors and increases their social status and privileges" (p. 137). | Discussion |
| 174 | Phatoli R, Bila N, Ross E. Being black in a white skin: Beliefs and stereotypes around albinism at a South African university, African Journal of Disability. 2015;4:106. http://dx.doi.org/10.4102/ajod.v4i1.106 | Social Development, Social Work | South Africa | South Africa | "... to explore the beliefs and practices regarding albinism within a South African university, and the availability of support services" (p. 1). | Qualitative |
| 53 | Pooe-Monyemore MB. A model for enhancement of self-concept of people with albinism (Doctoral thesis). https://uir.unisa.ac.za/bitstream/handle/10500/3914/thesis_pooe_b.pdf?sequence=1 (2007). Accessed 24 Jan 2018. | Health Studies | South Africa | Gauteng Province in South Africa | "… The main purpose of this study was to develop and describe a model for enhancement of self-concept of people with oculocutaneous albinism based on their life-experience" (p. 4). | Qualitative |
| 240 | Possi MK. Stigma and people with disability: A review of stigma attached to people with albinism. International Journal of Special Education. 1998;13:2. | Education | Tanzania | USA, South Africa, Zimbabwe, Nigeria, and Tanzania | "This paper attempts to address the problem of stigma of people with albinism and its implications" (p. 82). It focuses on defining albinism, stigma, exploring reasons for stigma against PWA as well as implications of stigma for PWA and their family. | Discussion |
| 185 | Possi MK, Milinga JR. Perceptions on people with albinism in urban Tanzania: Implications for social inclusion. Journal of Advocacy, Research and Education. 2018;5:2. | Education | Tanzania | Tanzania | "This article analyses the perceptions of people from urban Tanzania about individuals with  albinism. It attempts to evaluate people’s understanding of albinism, their attitudes towards individuals with the condition, and perceived reasons for their inhumane treatment" (p. 81). | Qualitative |
| 139 | Possi A, Possi A. The identity question versus appropriateness of legal anti-discrimination measures: Endorsing the disability rights approach to albinism. African Disability Rights Yearbook. 2017;5:118–140. | Law | Tanzania | International (although focusing on African countries) | "… While it is not in doubt that albinism currently is a human rights concern, the question of the ‘appropriate legal approach’ for realising the rights of persons with albinism appears not to have been conclusively answered. Thus, this contribution investigates the appropriate human rights approach for safeguarding the rights of persons with albinism and endorses the view that the disability rights approach appropriately accommodates matters concerning the rights and equality of persons with albinism" (p. 121). | Discussion |
| 10 | Reimer‑Kirkham S, Astle B, Ero I, Imafidon E, Strobell E. Mothering, albinism and human rights: The disproportionate impact of health-related stigma in Tanzania. Foundations of Science. 2020. https://doi.org/10.1007/s10699-020-09701-0 | (1,2, 5) Nursing, (3) Lawyer/ HR Advocacy (4) African Philosophy | Canada | Tanzania | "In a critical ethnographic study in Tanzania, we engaged with the voices of mothers impacted by albinism and key stakeholders to elucidate experiences of stigma" (p. 1). | Qualitative |
| 12 | Reimer-Kirkham S, Astle B, Ero I, Panchuk K, Dixon D. Albinism, spiritual and cultural practices, and implications for health, healthcare, and human rights: A scoping review. Disability & Society. 2019;34:5. https://doi.org/10.1080/09687599.2019.1566051 | (1,2,4) Nursing, (3) Lawyer/ HR Advocacy, (5) Librarian | International but focused on African context | International but focused on African context | "The purpose of this scoping review was to establish the current state of knowledge on albinism, spiritual and cultural practices, and implications for health and health services. Electronic searches of nine databases were conducted" (p. 747). | Systematic Review |
| 140 | Robertson VA. The racial pharmakon: Investigating albinism in African American literature (Doctoral dissertation). https://etd.ohiolink.edu/apexprod/rws_etd/send_file/send?accession=miami1146224861&disposition=inline (2006). 24 Jan 2018. | English | United States | United States (largely) | "This dissertation examines the manner in which black albinism circumvents the black/white binary opposition of Western racial discourse by simultaneously evoking two mutually exclusive racial identities. While phenotypically an African American, his blond hair, light eyes and fair skin make him whiter than most Caucasians" (Abstract). Specifically, "The overarching goal of this dissertation is to investigate the epiphany that race is a historical and social construction, to interrogate the problematic condition of the albinic body in the paradigmatic racial arena of black and white, and finally to explore the fluidity of albinism as an emancipatory tool of liberation" (p. 24). | Discussion |
| 175 | Robertson VA. The great white hope: Black albinism and the deposing of the white subject in John Edgar Wideman’s Sent For You Yesterday. Studies in Literature and Language. 2011;3:1-10. http://dx.doi.org/10.3968/n | English | USA | Events of the book take place in the USA | To critically analyze the symbolism of the albinic character, Brother Tate, in John Edgar Wideman's Sent For You Yesterday (1983). | Discussion |
| 141 | Schühle J. Medicine murder of people with albinism in Tanzania – how casino capitalism creates rumorscapes and occult economies. Centre for Area Studies Working Paper Series. 2013;2:3–35. | Social and Cultural Anthropology and Art History | Germany | Northwest Tanzania | "The news coverage rarely dug deeper to investigate the underlying problems behind this retreat to violence. Likewise, medical or sociological literature on albinism in Africa and especially in Tanzania is scarce. In 2009, I set out to conduct fieldwork in Northwest Tanzania in order to thoroughly analyze the murders in the most affected area" (p. 4). | Qualitative |
| 142 | Selepe DM. Teenagers with oculocutaneous albinism in Polokwane: Their self-esteem and perceptions of societal attitudes (Master’s thesis). http://ulspace.ul.ac.za/bitstream/handle/10386/97/M.A.%20Thesis%20_Selepe%20D.M_.pdf?sequence=3 (2007). Accessed 24 Jan 2018. | Psychology | Unclear | "...areas around Capricorn District of the Polokwane Municipality, Limpopo Province, South Africa" (p. 4). | "The aim of this study is to explore the self-esteem of teenagers with oculocutaneous albinism and perceived societal attitudes towards them" (p. 3). | Mixed |
| 80 | Strobell E. Exploring the experience of mothers who have children with albinism in Tanzania: A critical ethnography (Masters thesis). https://dam-oclc.bac-lac.gc.ca/download?is_thesis=1&oclc_number=1204228175&id=0a3d99a2-f4ca-4bff-966c-f0d1c1871cc2&fileName=file.pdf (2020). Accessed 22 Mar 2020. | Nursing | Canada | Tanzania | "A focused critical ethnographic study, through the lens of Hudson-Weems’ (2019) Africana Womanism, explored the experiences of mothers of children with albinism in Tanzania, addressed the gendered nature of this condition, and considered the human rights and resilience of the mothers" (p. 7). | Qualitative |
| 219 | Stroeken K. Stigmatized bodies near Lake Victoria: A cultural analysis of institutions. Foundations of Science. 2020. https://doi.org/10.1007/s10699-020-09684-y | Anthropology | Belgium | Tanzania | "This article concentrates on the most targeted region, Sukuma-speaking communities in Tanzania, to verify the stigmatizing impact of institutions: whether bride wealth treats women as commodities, whether children with nsebu disorder are stigmatized, and why children living with albinism are stigmatized" (p. 1). | Discussion |
| 241 | Tanner R. Ideology and the killing of albinos in Tanzania: A study in cultural relativities. Anthropologists. 2010;12:4. https://doi.org/10.1080/09720073.2010.11891161 | Anthropology | The Netherlands | North-west Tanzania | To explore why a cultural lens must be applied in the attempt to understand the behaviour of the Sukuma in albino killings: "This Sukuma violence should be understood in the context of inexplicable distress and within the accepted paradigm of widespread creatable spiritual power to do and prevent interpersonal evil" (p. 229). | Discussion |
| 11 | Taylor J, Bradbury-Jones C, Lund P. Witchcraft-related abuse and murder of children with albinism in Sub-Saharan Africa: A conceptual review. Child Abuse Review. 2019;28:13–26. https://doi.org/10.1002/car.2549 | (1,2) Institute of clinical sciences, (3) Geneticist | England | Sub-Saharan Africa | "This conceptual review summarises the scarce research on the topic of children with albinism within an integrated framework of otherness (highlighting the ways in which these children differ from others in their group and how this is perceived by themselves and others), watchfulness (the consequences and impact of this very visible difference on families and communities) and agency (what such children themselves and other people can do to respond to these abuses of human rights)" (p. 13). | Systematic Review |
| 54 | Taylor JS, Lund P. Experiences of a feasibility study of children with albinism in Zimbabwe: A discussion paper. International Journal of Nursing Studies. 2007;45:8. https://doi.org/10.1016/j.ijnurstu.2007.05.009 | Nursing; Midwifery; Biomolecular and Sport Sciences | United Kingdom | Zimbabwe (Bulawayo, the second largest city in Zimbabwe located in the southern part of the country in Matabeleland p. 1251-1252). | "The technical, economic, legal, collaborative, operational, schedule and political feasibilities (acronym TELCOSP) to undertaking a study on children with albinism [specifically, their psychosocial and welfare needs] in Zimbabwe were explored over a six week period of ﬁeldwork in the country" (p. 1247). -- "the focus of this paper is to explore the challenges and opportunities of undertaking international research in challenging settings" (p. 1248). | Discussion |
| 143 | Thurston M. “They think they know what’s best for me”: An interpretative phenomenological analysis of the experience of inclusion and support in high school for vision-impaired students with albinism. International Journal of Disability, Development and Education. 2014;61:2. http://dx.doi.org/10.1080/1034912X.2014.905054 | Social and Health Sciences | United Kingdom | United Kingdom (Britain) | "This study used interpretative phenomenological analysis to examine and understand the way in which two vision-impaired students with albinism experienced inclusion and support in high school" (p. 108). | Qualitative |
| 242 | TuSmith B. The “inscrutable albino” in contemporary ethnic literature. Amerasia Journal. 1993;19:3. https://doi.org/10.17953/amer.19.3.l0373kv41436831j | English | United States | United States (inferred) | ".... the present discussion will focus on some provocative aspects of a specific literary trope: the mysterious figure of the "inscrutable albino"" (p. 85). Specifically, "... to explore the albino motif in a few sample ethnic works to ascertain how the trope functions within each work and what this says about the world-view embedded in the text" (p. 87). | Discussion |
| 55 | Udeh NN, Eze BI, Onwubiko SN, Arinze OC, Onwasigwe EN, Umeh RE. Oculocutaneous albinism: Identifying and overcoming barriers to vision care in Nigerian population. Journal of Community Health. 2014;39:508-503. https://doi.org/10.1007/s10900-013-9787-5 | Ophthalmology | Nigeria | Enugu state, South-East Nigeria | "To assess eye care service utilization, and identify access barriers in a south-eastern Nigerian albino population" (p. 508). | Cross-sectional |
| 56 | Uromi SM. Violence against persons with albinism and older women: tackling witchcraft accusations in Tanzania. International Journal of Education and Research. 2014;2:6. https://ijern.com/journal/June-2014/25.pdf | Finance | Tanzania | Tanzania | The author focuses on exploring violations to PWAs' rights, and on offering recommendations for how those rights may instead be upheld. | Discussion |
| 144 | Vander Kolk GJ, Bright BC. Albinism: A survey of attitudes and behavior. Journal of Visual Impairment & Blindness. 1983;77:49. https://doi.org/10.1177/0145482X8307700201 | Rehabilitation Services | United States | None per se; respondents were from the US and the UK, but reference was also made to, for example, Africa | "Surveys the general public's knowledge of and attitudes toward albinism and shows how children's attitudes can be changed" (p. 48). | Qualitative |
| 243 | Wambu O. The science and magical realism of killing albinos: Is there really a difference between the silent genocide against albinos and the quantum theory of all-night prayer sessions? https://www.questia.com/magazine/1G1-471553747/the-science-and-magical-realism-of-killing-albinos (2016). Accessed 24 Jan 2018. | Uncertain | Uncertain | None per se; killings in Southern Africa discussed. | To show that there is no justifiable reasoning behind albino killings. | Discussion |
| 244 | Wan-Kee-Chaung N. The voices of albinism (Master’s thesis). https://spectrum.library.concordia.ca/id/eprint/1667/1/MQ68394.pdf (2001). Accessed 24 Jan 2018. | Sociology and Anthropology | Canada (Concordia University) | None in particular; respondents were from Canada, the US, and Australia. | "This thesis proposes to give voice to [PWA] as they recount the negative social repercussions they face in societies that marginalize and stigmatize people with an unconventional physical appearance and a disability, and also how they cope with such discrimination and prejudice" (p. 4)... "Focus is placed on the strategies that respondents have devised in coping with the adversities that often accompany a highly visible physical difference and disability" (p. iii). | Qualitative |
| 176 | Westhoff W. A psychosocial study of albinism in a predominantly Mulatto Caribbean community. Psychological Reports. 1993;73:1007-1010. https://doi.org/10.1177/00332941930733pt143 | Public Health | United States | Dominican Republic | "The purpose of this study was to collect data on albinos to test the hypothesis that albinism is a stigma and can create a negative separation between the individual and the community. The author was also interested in knowing if albinism contributed to psychosocial problems for the individual" (p. 1008). | Qualitative |
| 57 | White T. ‘Their whiteness is not like ours’: A social and cultural history of albinism and albino identities, 1650-1914 (Doctoral dissertation). https://www.proquest.com/openview/563f38902e8463eca3e9e471a8d83abd/1?pq-origsite=gscholar&cbl=51922 (2011). Accessed 24 Jan 2018. | Humanities (Art, History and Cultures) | England (University of Manchester) | Examining albinism from a medical model perspective in "modern Europe and the United States" (p. 18); however, mentions other countries worldwide in which Europeans interacted with persons with albinism. | “This thesis traces the history of albinism back to the first early modern interactions between medicine, science and people with an unusually white complexion. It investigates the relationship between medico-scientific definitions of albinism and the diagnosed individual. It tracks the formation of albinism as medical category in European and North American medicine and the life sciences. It weighs up how albinism and the ‘albino’ contributed to its inclusion in wider medical and social debates about ability, disability and what Catherine Hall calls the ‘unspoken norm’ of whiteness in nineteenth and early twentieth century western culture and society.4 It locates albinism as an intermittently constructed biological condition, mediated by social conceptions of normal and abnormal whiteness. It ultimately seeks out the reasons why this unusually white bodily condition and its accompanying visual impairment ignited such sustained and universal interest among explorers, philosophers, scientists and medical practitioners from the seventeenth century to the start of the First World War. It analyses the long history of albinism to shed new light on the troubling dichotomies of race and skin colour, normalcy and difference and medical power and agency" (p. 11-12). | Discussion |
| 58 | Wright CY, Norval M, Hertle RW. Oculocutaneous albinism in Sub‐Saharan Africa: Adverse sun‐associated health effects and photoprotection. Photochemistry and Photobiology. 2015;91:1. https://doi.org/10.1111/php.12359 | Climate Studies (Primary author); Geography, Geoinformatics and Meteorology;  Biomedical Sciences;  Vision;  Surgery | South Africa (Primary author) | Sub-Saharan Africa | "In this review, we identify the characteristics and prevalence of OCA in sub-Saharan Africa and describe the ﬁndings of studies that have assessed sun exposure patterns and photoprotection use by individuals with OCA. The challenges and opportunities toward the optimal means of photoprotection for these at-risk individuals in the context of sub-Saharan Africa are identiﬁed" (p. 27). | Systematic Review |

| **Reference Number** | **GREY LITERATURE: Title of Articles** | **Location of author(s)** | **Type** |
| --- | --- | --- | --- |
| 103 | ADD International: Impact report: Women and girls edition. https://www.add.org.uk/sites/default/files/2017_ImpactReport_Spring.pdf (2017). Accessed 18 Feb 2018. | Tanzania, Uganda | NGO |
| 186 | African Commission on Human and Peoples’ Rights: Resolution on the regional action plan on albinism in Africa (2017-2021) - ACHPR/Res. 373 (LX) 2017. https://www.acdhrs.org/wp-content/uploads/2017/07/Resolution-on-the-Regional-Action-Plan-on-Albinism-in-Africa.pdf (2017). Accessed 18 Feb 2018. | Nigeria | Intergovernmental organization (IGO) |
| 193 | Amnesty International: Malawi: Towards effective criminal justice for people with albinism in Malawi. https://www.amnesty.org/en/documents/afr36/8634/2018/en/ (2018). Accessed 18 Feb 2018. | International | NGO |
| 90 | Amnesty International: People with albinism in Malawi: Item 3 Amnesty International’s written statement on the 34th session of the UN Human Rights Council (27 February- 24 March 2017). https://reliefweb.int/sites/reliefweb.int/files/resources/AFR3656762017ENGLISH.pdf (2017). Accessed 18 Feb 2018. | International | NGO |
| 92 | Amnesty International: “We are not animals to be hunted or sold”: Violence and discrimination against people with albinism in Malawi. https://www.amnesty.org/en/documents/afr36/4126/2016/en/ (2016). Accessed 18 Feb 2018. | International | NGO |
| 156 | Christian Blind Mission (CBM): Security guideline for people with albinism: Concrete and specific security measures to prevent and handle attacks on people with albinism. https://www.medbox.org/document/security-guideline-for-people-with-albinism-concrete-and-specific-security-measures-to-prevent-and-handle-attacks-on-people-with-albinism#GO (2017). Accessed 18 Feb 2018. | Germany | NGO |
| 96 | Commission for Human Rights and Good Governance (CHRAGG): Individual report of the Tanzania national human rights institution submission to the human rights council. https://lib.ohchr.org/HRBodies/UPR/Documents/session12/TZ/CHRAGG-CommissionHumanRightsGoodGovernance-eng.pdf (2011). Accessed 18 Feb 2018. | Tanzania | Government |
| 95 | European Parliament: Situation of persons with albinism in Africa, notably in Malawi. https://eur-lex.europa.eu/legal-content/EN/TXT/PDF/?uri=CELEX:52016IP0314&from=ET (2016). Accessed 18 Feb 2018. | Europe | Government |
| 111 | European Parliament: Situation of people with albinism in Malawi and other African countries. https://www.europarl.europa.eu/doceo/document/TA-8-2017-0381_EN.html (2017). Accessed 18 2018. | Europe | Government |
| 188 | Federal Ministry of Education: Implementation of guidelines. https://albinofoundation.org/wp-content/uploads/2017/04/National-Policy-on-Albinism-Implementatio-Guideline.pdf (2012). Accessed 18 Feb 2018. | Nigeria | Government |
| 245 | Federal Ministry of Education: National policy on albinism in Nigeria: Implementation of guidelines. https://albinofoundation.org/wp-content/uploads/2017/04/National-Policy-on-Albinism-Implementatio-Guideline.pdf (2012). Accessed 18 Feb 2018. | Nigeria | Government |
| 206 | Foundation for Civil Society: Annual report 2017: Strengthening Tanzania's civil society to empower citizens. https://thefoundation.or.tz/download/2017-annual-report/ (2017). Accessed 18 Feb 2018. | Tanzania | NGO |
| 160 | Fraser E, Legwegoh A, KC K, Davis M: Toil and trouble: How conflict and climate change are triggering witch hunts. Foreign Affairs. https://www.foreignaffairs.com/articles/2015-08-16/toil-and-trouble (2015). Accessed 18 Feb 2018. | Canada | Media |
| 246 | Human Rights Watch: World report 2018 - Mozambique. https://www.refworld.org/docid/5a61ee49a.html (2018). Accessed 10 April 2020. | International | NGO |
| 105 | International Federation of Red Cross and Red Crescent Societies: Through albino eyes: The plight of albino people in Africa’s Great Lakes region and a Red Cross response –advocacy report. https://www.ifrc.org/Global/Publications/general/177800-Albinos-Report-EN.pdf (2009). Accessed 18 Feb 2018. | Switzerland | NGO |
| 200 | International Organization for Migration: Situation analysis on the human rights and protection of persons with albinism (PWA) from human trafficking and discrimination in Mozambique. https://mozambique.iom.int/sites/default/files/Vacancy/document/TOR_IOM%20Mozambique_Situation%20Analysis%20PwA%20%28FINAL%29_23.05.2018.pdf (2018). Accessed 10 April 2020. | International | IGO |
| 100 | Lund P, Betts J, Beale J: An investigation into the impact of stigma on the education and life opportunities available to children and young people with albinism in Tanzania and Uganda. Final report. International Foundation of Applied Disability Research. https://www.firah.org/upload/l-appel-a-projets/projets-laureats/2018/albinisme/coventry-university-ap2014_47-firah-final-report-def.pdf (2018). Accessed 18 Feb 2018. | UK, Tanzania | NGO |
| 207 | Malawi Human Rights Commission: Media release: Commemoration of 2019 international albinism awareness day. http://mhrcmw.org/mhrc/sites/default/files/Media%20Release%20on%20Commemoration%20of%202019%20International%20Albinism%20Awareness%20Day%2C%20June%202019.pdf (2019). Accessed 10 April 2020. | Malawi | Government |
| 201 | Mennonite Central Committee of Tanzania (MCC): Changing lives of people with albinism in Tanzania MCC progress report. https://rotary-site.org/resources/389/files/CHANGING%20THE%20LIVES%20OF%20ALBINOS%20IN%20TANZANIA%20-%20PROGRESS%20REPORT.pdf (2015). 18 Feb 2018. | Tanzania | NGO |
| 163 | Ministry of Gender, Disabilities and Social Welfare: Malawi country report. https://archive.uneca.org/sites/default/files/uploaded-documents/Beijing25/malawi-beijing25_report.pdf (2019). Accessed 10 April 2020. | Malawi | Government |
| 162 | Office of the High Commissioner of Human Rights (OHCHR): Committee on the Rights of Persons with Disabilities opens seventeenth session. https://www.ohchr.org/EN/NewsEvents/Pages/DisplayNews.aspx?NewsID=21425&LangID=E (2017). Accessed 18 Feb 2018. | International | IGO |
| 216 | Office of the High Commissioner of Human Rights (OHCHR): Regional action plan on albinism in Africa 2 2017 to 2021: 5-year plan to address attacks and related violations against persons with albinism in Sub Saharan Africa. https://www.hrfn.org/wp-content/uploads/2017/10/RegionalAction_Plan_on_albinism_EN.pdf (2016). Accessed 18 Feb 2018. | International | IGO |
| 187 | Permanent Mission of the Kingdom of Swaziland. The human rights situation of people living with albinism in the Kingdom of Swaziland. https://www.ohchr.org/Documents/HRBodies/HRCouncil/AdvisoryCom/Albinism/Swazilland.pdf (2014). Accessed 18 Feb 2018. | Swaziland | government |
| 91 | Parliament of South Africa: Report of the select committee of public participation, petitions and members’ legislative proposal on parliament for people with albinism held on the 21st June 2017 Mpumalanga provincial legislature members. http://www.mpuleg.gov.za/assets/atc-147-2017--report-people-with-albinism.pdf (2017). Accessed 18 Feb 2018. | South Africa | Government |
| 112 | Republic of Malawi: Integrating COVID-19 messaging/ feedback collection in radio programs. (2020). Accessed 10 April 2020. | Malawi | Government |
| 162 | Rohwerder B: Disability stigma in the Disability Inclusive Development (DID) programme countries: An overview of the evidence. Institute of Development Studies. https://opendocs.ids.ac.uk/opendocs/handle/20.500.12413/15131 (2019). Accessed 10 April 2020. | UK | Academic |
| 194 | Semkwiji D: The plight of Albino in Tanzania: What should be done? The Economic and Social Research Foundation. http://www.esrftz.org/docs/ALBINO_POLICYBRIEF_04-2009%20(2).PDF (2009). Accessed 18 Feb 2018. | Tanzania | NGO |
| 97 | Shayo L: The role played by museums in protection persons with albinism in Tanzania. http://bibalex.org/baifa/en/resources/document/449539 (2010). Accessed 18 Feb 2018. | Tanzania | Government |
| 204 | South African Law Reform Commission: The review of the Witchcraft Suspension Act 3 of 1957. https://www.justice.gov.za/Salrc/dpapers/dp139-WitchcraftSuppression.pdf (2016). Accessed 18 Feb 2018. | South Africa | Government |
| 81 | Southern African Catholic Bishops’ Conference: Albinism in Africa. https://cisp.cachefly.net/assets/articles/attachments/48243_albinism_in_africa_dec_2013.pdf (2013). Accessed 18 Feb 2018. | Cape Town, Durban, Pretoria, Johannesburg and Bloemfontein | NGO |
| 203 | Standing Voice: Evaluation report: Science, art, community building initiative understanding of albinism in Tanzania. https://media.tghn.org/articles/Standing_Voice_Evaluation_Report.pdf (2018). Accessed 10 April 2020. | Tanzania, Malawi | NGO |
| 205 | The Albino Foundation (TAF): Activity report of the international albinism awareness day celebration. https://albinofoundation.org/wp-content/uploads/2017/03/Report-on-2015-International-Albinism-Day-Celebration.pdf (2015). Accessed 18 Feb 2018. | Nigeria | NGO |
| 189 | The Albino Foundation (TAF): Report of a two day public workshop on albinism, held by The Albino Foundation (TAF) Enugu Chapter. https://albinofoundation.org/wp-content/uploads/2017/03/Enugu-Programme-Report.pdf (2013). Accessed 18 Feb 2018. | Nigeria | NGO |
| 153 | The Albino Foundation (TAF): Report of The Albino Foundation sensitisation workshop for judges/ magistrates, lawyers and law enforcement agencies in FCT on the protection and enforcement of the rights of persons with albinism held on the 5th of December 2017 at the FCT High Court, Abuja. https://albinofoundation.org/wp-content/uploads/2017/12/LAW-ENFORCEMENT-AGENCIES-SENSITISATION-WORKSHOP-1.pdf (2017). Accessed 18 Feb 2018. | Nigeria | NGO |
| 154 | The Albino Foundation (TAF): Report of The Albino Foundation sensitization workshop for principals of secondary schools in FCT on the teaching of children with albinism in school held on the 30th of November 2017 at the British Council. https://albinofoundation.org/wp-content/uploads/2018/02/The-Albino-Foundation-2017-Annual-Report51067-3-min.pdf (2017). Accessed 18 Feb 2018. | Nigeria | NGO |
| 84 | The Albino Foundation (TAF): Report of The Albino Foundation’s albinism rights project launch and workshop for its coordinators within the north central region of Nigeria held on the 12th and 13th of September 2017. https://albinofoundation.org/wp-content/uploads/2017/12/ADVOCACY-AND-PWA-CAPACITY-BUILDING-REPORT-1.pdf (2017). Accessed 18 Feb 2018. | Nigeria | NGO |
| 205 | The Albino Foundation (TAF): Report of the 2017 international albinism awareness day celebration held on the 13th of June 2017 at the National Human Rights Commission, Abuja Nigeria. http://albinofoundation.org/wp-content/uploads/2017/03/2017-TAF-IAAD-report.pdf (2017). Accessed 18 Feb 2018. | Nigeria | NGO |
| 85 | The Albino Foundation (TAF): Strategic plan document for The Albino Foundation (2016-2020). https://albinofoundation.org/wp-content/uploads/2017/04/TAF-STRATEGIC-PLAN-2016-2020.pdf (2016). Accessed 18 Feb 2018. | Nigeria | NGO |
| 87 | The Albino Foundation (TAF): The Albino Foundation annual report 2013: Committed… to fight the cause of albinism. https://albinofoundation.org/wp-content/uploads/2017/03/TAF-2013-Annual-Report-2.pdf (2013). Accessed 18 Feb 2018. | Nigeria | NGO |
| 192 | The Albino Foundation (TAF): The Albino Foundation 2014 annual report. https://albinofoundation.org/wp-content/uploads/2017/03/TAF-2014-ANNUAL-REPORT.pdf (2014). Accessed 18 Feb 2018. | Nigeria | NGO |
| 191 | The Albino Foundation (TAF): The Albino Foundation 2015 annual report. https://albinofoundation.org/wp-content/uploads/2017/03/TAF-2015-Annual-Report.pdf (2015). Accessed 18 Feb 2018. | Nigeria | NGO |
| 82 | The Albino Foundation (TAF): The demographic, geographic and socioeconomic survey of persons with albinism in Nigeria. https://actiononalbinism.org/fr/document/3a3i9umalw3zd771xkdoq1tt9?page=9 (2018). Accessed 10 April 2020. | Nigeria | NGO |
| 83 | The Albino Foundation (TAF): 2017 annual report. https://albinofoundation.org/wp-content/uploads/2018/02/The-Albino-Foundation-2017-Annual-Report51067-3-min.pdf (2017). Accessed 18 Feb 2018. | Nigeria | NGO |
| 195 | The Pan-African Parliament: Resolutions on persons with albinism in Africa. https://www.chr.up.ac.za/images/centrenews/2018/files/2018_pap_resolution_on_persons_with_albinism.pdf (2018). Accessed 10 April 2020. | South Africa | Government |
| 102 | Uganda Project Implementation and Management Centre (UPIMAC): Albinism proposal: Improving the lives of people with albinism in the communities of Jinja, Mayuge and Buyende Districts in Busoga Sub region. https://www.globalgiving.org/pfil/26552/projdoc.pdf (2016). Accessed 18 Feb 2018. | Uganda | NGO |
| 157 | Under the Same Sun (UTSS): Clarifying albinism: Transforming perceptions & ushering in protection with the help of international human rights law. http://globaldisability.org/wp-content/uploads/2016/01/Executive-Summary-on-PWA-Classification.pdf (2014). Accessed 18 Feb 2018. | Canada and Tanzania | NGO |
| 158 | Under the Same Sun (UTSS): Discrimination against women and girls with albinism in Malawi. https://tbinternet.ohchr.org/Treaties/CEDAW/Shared%20Documents/MWI/INT_CEDAW_NGO_MWI_22043_E.pdf (2015). Accessed 18 Feb 2018. | Tanzania | NGO |
| 197 | Under the Same Sun (UTSS): History of attacks against persons with albinism (PWA). https://pdfs.semanticscholar.org/f2ca/6ea1f4509ca0069f8a1e34838cbc4b4786c6.pdf (2013). Accessed 18 Feb 2018. | Canada | NGO |
| 94 | Under the Same Sun (UTSS): Kenyans with albinism and racial discrimination. https://tbinternet.ohchr.org/Treaties/CERD/Shared%20Documents/KEN/INT_CERD_NGO_KEN_27123_E.pdf (2017). Accessed 18 Feb 2018. | Kenya | NGO |
| 161 | Under the Same Sun (UTSS): Recommended actions for the Tanzanian government: An adequate response to persons with albinism in Tanzania. http://globaldisability.org/wp-content/uploads/2016/01/Recommended-Actions-for-TZ-Govt.pdf (2016). Accessed 18 Feb 2018. | Tanzania | NGO |
| 159 | Under the Same Sun (UTSS): The political and civil rights of people with albinism in the Democratic Republic of Congo. https://www.ecoi.net/en/file/local/1399027/1930_1493285932_int-ccpr-ico-cod-26791-e.pdf (2017). Accessed 18 Feb 2018. | Congo | NGO |
| 86 | UNICEF: Knowledge attitude and practices: Study on children with albinism in Nigeria. https://albinofoundation.org/wp-content/uploads/2017/03/UNICEF-Report-on-Children-with-albinism-in-Nigeria.pdf (2017). Accessed 18 Feb 2018. | International | NGO |
| 113 | UNICEF: 2018/19 disability budget brief. https://www.unicef.org/esa/sites/unicef.org.esa/files/2019-04/UNICEF-Malawi-2018-Disability-Budget-Brief.pdf (2019). Accessed 10 April 2020. | International | IGO |
| 106 | United Nations National Assembly (UNGA): Applicable international human rights standards and related obligations addressing the issues faced by persons with albinism (A/72/131). https://documents-dds-ny.un.org/doc/UNDOC/GEN/N17/214/51/PDF/N1721451.pdf?OpenElement (2017). Accessed 18 Feb 2018. | International | IGO |
| 117 | United Nations General Assembly (UNGA): Corrigendum to the albinism worldwide report (A/74/190/Corr.1). https://undocs.org/A/74/190/Corr.1 (2019). Accessed 10 April 2020. | International | IGO |
| 116 | United Nations General Assembly (UNGA): Country visit report to Fiji (A/HRC/40/62/Add.1). https://ap.ohchr.org/documents/dpage_e.aspx?si=A/HRC/40/62/Add.1 (2019). Accessed 10 April 2020. | International | IGO |
| 124 | United Nations General Assembly (UNGA): Country visit report to Kenya (A/HRC/40/62/Add.3). https://ap.ohchr.org/documents/dpage_e.aspx?si=A/HRC/40/62/Add.3 (2018). Accessed 10 April 2020. | International | IGO |
| 119 | United Nations General Assembly (UNGA): Country visit report to the republic of South Africa (A/HRC/43/42/Add.1). https://undocs.org/A/HRC/43/42/Add.1 (2020). Accessed 10 April 2020. | International | IGO |
| 196 | United Nations General Assembly (UNGA): International albinism awareness day. https://digitallibrary.un.org/record/782921?ln=en (2014). Accessed 18 Feb 2018. | International | IGO |
| 199 | United Nations General Assembly (UNGA): Persons with albinism (A/C.3/70/L.14/Rev.1). https://documents-dds-ny.un.org/doc/UNDOC/GEN/N15/377/98/PDF/N1537798.pdf?OpenElement (2015). Accessed 18 Feb 2018. | International | IGO |
| 123 | United Nations General Assembly (UNGA): Preliminary findings of country visit to Kenya by the United Nations Independent Expert on the enjoyment of human rights by persons with albinism. https://www.ohchr.org/Documents/Issues/Albinism/PreliminaryfindingsKenya_EN.docx (2018). Accessed 10 April 2020. | International | IGO |
| 107 | United Nations General Assembly (UNGA): Preliminary survey on the root causes of attacks and discrimination against persons with albinism (A/71/255). https://documents-dds-ny.un.org/doc/UNDOC/GEN/N16/241/46/PDF/N1624146.pdf?OpenElement (2016). Accessed 18 Feb 2018. | International | IGO |
| 99 | United Nations General Assembly (UNGA): Report of the Human Rights Council Advisory Committee on the study on the situation of human rights of persons living with albinism (A/HRC/28/75). https://ap.ohchr.org/documents/dpage_e.aspx?si=A%2FHRC%2F28%2F75 (2015). Accessed 18 Feb 2018. | International | IGO |
| 109 | United Nations General Assembly (UNGA): Report of the Independent Expert on the enjoyment of human rights by persons with albinism on her mission to Malawi (A/HRC/34/59/Add.1). https://documents-dds-ny.un.org/doc/UNDOC/GEN/G16/433/28/PDF/G1643328.pdf?OpenElement (2016). Accessed 18 Feb 2018. | International | IGO |
| 110 | United Nations General Assembly (UNGA): Report of the Independent Expert on the enjoyment of human rights by persons with albinism on her mission to Mozambique (A/HRC/34/59/Add.2). https://documents-dds-ny.un.org/doc/UNDOC/GEN/G16/433/59/PDF/G1643359.pdf?OpenElement (2016). Accessed 18 Feb 2018. | International | IGO |
| 89 | United Nations General Assembly (UNGA): Report of the Independent Expert on the enjoyment of human rights by persons with albinism on her mission to the United Republic of Tanzania (A/HRC/37/57/Add.1). https://documents-dds-ny.un.org/doc/UNDOC/GEN/G17/364/15/PDF/G1736415.pdf?OpenElement (2017). Accessed 18 Feb 2018. | International | IGO |
| 108 | United Nations General Assembly (UNGA): Report of the Independent Expert on the enjoyment of human rights by persons with albinism on the expert workshop on witchcraft and human rights (A/HRC/37/57/Add.2). https://documents-dds-ny.un.org/doc/UNDOC/GEN/G18/016/97/PDF/G1801697.pdf?OpenElement (2018). Accessed 10 April 2020. | International | IGO |
| 217 | United Nations General Assembly (UNGA): Report of the Independent Expert on the enjoyment of human rights by persons with albinism on the Regional Action Plan on Albinism in Africa (2017–2021) (A/HRC/37/57/Add.3). https://documents-dds-ny.un.org/doc/UNDOC/GEN/G17/363/74/PDF/G1736374.pdf?OpenElement (2017). Accessed 18 Feb 2018. | International | IGO |
| 101 | United Nations General Assembly (UNGA): Right of persons with albinism to the highest attainable standard of health (A/HRC/37/57). https://documents-dds-ny.un.org/doc/UNDOC/GEN/G17/364/80/PDF/G1736480.pdf?OpenElement (2018). Accessed 10 April 2020. | International | IGO |
| 27 | United Nations General Assembly (UNGA): Round table on human rights and albinism: seeking consensus and priorities on advocacy and research: Report of the Independent Expert on the enjoyment of human rights by persons with albinism (A/HRC/40/62/Add.2). https://ap.ohchr.org/documents/dpage_e.aspx?si=A/HRC/40/62/Add.2 (2019). Accessed 10 April 2020. | International | IGO |
| 114 | United Nations General Assembly (UNGA): The 2030 agenda for sustainable development and the human rights of persons with albinism (A/73/181). https://ap.ohchr.org/documents/dpage_e.aspx?si=A/73/181 (2018). Accessed 10 April 2020. | International | IGO |
| 118 | United Nations General Assembly (UNGA): Thematic report: Albinism worldwide (A/74/190). https://undocs.org/A/74/190 (2019). Accessed 10 April 2020. | International | IGO |
| 115 | United Nations General Assembly (UNGA): Thematic report on access to justice (A/HRC/40/62). https://ap.ohchr.org/documents/dpage_e.aspx?si=A/HRC/40/62 (2019). Accessed 10 April 2020. | International | IGO |
| 122 | United Nations General Assembly (UNGA): Thematic report: Protection of persons with albinism (A/75/170). https://undocs.org/A/75/170 (2020). Accessed 10 April 2020. | International | IGO |
| 120 | United Nations General Assembly (UNGA): Thematic report: Women and children impacted by albinism (A/HRC/43/42). https://undocs.org/A/HRC/43/42 (2020). Accessed 10 April 2020. | International | IGO |
| 98 | United Nations General Assembly (UNGA): Vision of the mandate (A/HRC/31/63). https://documents-dds-ny.un.org/doc/UNDOC/GEN/G16/007/00/PDF/G1600700.pdf?OpenElement (2016). Accessed 18 Feb 2018. | International | IGO |
| 3 | United Nations General Assembly (UNGA): Witchcraft and the human rights of persons with albinism (A/HRC/34/59). https://documents-dds-ny.un.org/doc/UNDOC/GEN/G17/004/01/PDF/G1700401.pdf?OpenElement (2017). Accessed 18 Feb 2018. | International | IGO |
| 121 | United Nations Human Rights Special Procedures: Best practices in the protection of human rights by persons with albinism. https://www.ohchr.org/Documents/Issues/Albinism/A-75-170-Addendum.pdf (2020). Accessed 10 April 2020. | International | IGO |
| 202 | United Nations Office of the Special Representative of the Secretary on Violence against Children: Protecting children from harmful practices in plural legal systems: With special emphasis on Africa. https://violenceagainstchildren.un.org/news/protecting-children-harmful-practices-plural-legal-systems-special-emphasis-africa-0 (2012). Accessed 18 Feb 2018. | International | IGO |
| 210 | United Nations Partnerships on the Rights of Persons with Disabilities (UNPRPD): UNPRPD fund 2018 annual report. https://unprpd.org/sites/default/files/library/2019-06/UNPRPD%202018%20Annual%20Narrative%20Report.pdf (2018). Accessed 10 April 2020. | International | IGO |
| 93 | United States Department of State: Tanzania 2017 human rights report. https://www.state.gov/report/custom/8fbf122280/ (2017). Accessed 18 Feb 2018. | Tanzania | Government |
| 104 | United States Department of State: 2015 county reports on human rights practices – Malawi. https://www.refworld.org/docid/5716123f15.html (2016). Accessed 18 Feb 2018. | Malawi | Government |
| 198 | Witchcraft & Human Rights Information Network (WHRIN): Exploring the role of Nollywood in the muti murders of persons with albinism. http://www.whrin.org/wp-content/uploads/2013/08/Exploring-the-Role-of-Nollywood-in-the-Muti-Murders-of-PWA-Report-for-the-UNOHCHR-FINAL.pdf (2013). Accessed 18 Feb 2018. | International | NGO |
| 190 | Witchcraft and Human Rights Information Network (WHRIN): Witchcraft accusations and persecution; Muti murders and human sacrifice: Harmful beliefs and practices behind a global crisis in human rights. http://www.whrin.org/wp-content/uploads/2017/10/2017-UNREPORT-final.pdf (2017). Accessed 18 Feb 2018. | International | NGO |
| 215 | Women, Youth and Persons with Disabilities. Message from Minister Maite Nkoana-Mashabane on the Occasion of the Exit Briefing by the UN Independent Expert on the Enjoyment of Human Rights by Persons with Albinism, Ms. Ikponwosa Ero. Republic of South Africa (2019). Accessed 10 April 2020. | South Africa | Government |
