# Supplementary material for: Mapping a research-advocacy-policy agenda on human rights and albinism: a mixed methods project
Source: Int J Equity Health. 2024 Jan 2;23:1. doi: 10.1186/s12939-023-02064-5 (PMC10762980; doi:10.1186/s12939-023-02064-5)
Supplement: Supplementary file 2 — Additional file 2. Supplementary Materials: Survey Items (Round 2). [file 12939_2023_2064_MOESM2_ESM.docx]

**SUPPLEMENTARY MATERIALS: SURVEY ITEMS (Round 2)**

| **ADVOCACY STRATEGIES** |
| --- |

**INTERNATIONAL ADVOCACY STRATEGIES**

1. Advocacy strategies originating from **international non-governmental organizations** for human rights of persons with albinism.
2. Advocacy strategies that implement and **monitor United Nations Conventions** that relate to persons with albinism (e.g. disability, women, children, human rights).
3. Advocacy strategies in which **countries** with influence over international governments **work to leverage their influence** in favour of the human rights of persons with albinism.
4. Advocacy strategies to secure **financial assistance from the international community** to support persons with albinism in the Global South.
5. Advocacy strategies to lobby for openness among **nations to receive persons with albinism as asylum seekers** (refugees) from countries in which they suffer human rights abuses.
6. Advocacy strategies to increase cooperation between adjacent nations to **prevent cross-border crimes** against persons with albinism.
7. Advocacy strategies to develop **international guidance on witchcraft and human rights** in particular to end witchcraft-related harmful practices (including, but not limited to, those practices affecting persons with albinism).

**INTERSECTORAL ADVOCACY STRATEGIES**

1. Advocacy that creates **strategic alliances among non-governmental organizations** to create more integrated and efficient advancements for persons with albinism.
2. Intersectoral advocacy strategies that primarily seek to **alleviate poverty**.
3. Promote the Worldwide **Albinism Awareness Day**.

**LOCAL ADVOCACY STRATEGIES**

1. Advocacy to improve access to **education** for persons with albinism.
2. Advocate for **job training** for persons with albinism to increase employability.
3. Advocacy to improve access to **disability benefits**.
4. Advocacy to improve **vision care** for persons with albinism.
5. Advocacy to improve **skin care** for persons with albinism.
6. Advocacy to improve access to comprehensive healthcare (**not just vision and skin care**) for persons with albinism.
7. Advocacy for access to **technology** that can improve the lives of people with albinism (e.g. computers with large letters and ability to zoom in).
8. Advocacy through strategic dialogue among **community groups**.
9. Advocacy strategies that use **public events and rallies** to demonstrate support for persons with albinism and encourage social and/or legal change.
10. Advocacy strategies that communicate factual messages about persons with albinism using **first-hand stories**.
11. Advocacy for curriculum revision to **educate all children** with accurate information about albinism from grade school onwards.
12. Advocacy strategies for monitoring and **exposing inaccurate media** **representations** of persons with albinism.
13. Advocacy strategies that create positive and realistic **movies and documentaries** about people with albinism, including people with albinism as characters.
14. Advocacy strategies that use **theatre** (plays) to communicate truths about albinism and dispel myths.
15. Advocacy strategies that use **radio** (songs, jingles, and talk shows) to communicate truths about albinism and dispel myths.
16. Advocacy strategies that use **literature** (novels, short stories, etc.) to create awareness about albinism.
17. Advocacy strategies that form a media campaign across **multiple forms of media** to create awareness about albinism.
18. Advocacy strategies that **publicize arrests** of persons who perpetrate violence against persons with albinism.
19. Advocacy strategies that provide **legal representation** for persons with albinism in cases pertaining to transgressions against their enjoyment of human rights.

**AVENUES OF INFLUENCE**

1. Advocacy strategies that aim to create change through the activities of the **United Nations**.
2. Advocacy strategies that aim to create change through the activities of the **African Union**.
3. Advocacy strategies that target primarily **national governments**.
4. Advocacy strategies that target primarily **local governments**.
5. Advocacy strategies that **seek alliances** with **supportive politicians**.
6. Advocacy strategies that involve **researchers** and academics.
7. Advocacy strategies that create, support, and make central the work of **albinism support groups**.
8. Advocacy strategies that target primarily **witchdoctors** and traditional healers.
9. Advocacy strategies that target primarily **buyers** of products created with the body parts of persons with albinism.
10. Advocacy strategies that target primarily **law enforcement personnel**.
11. Advocacy strategies that target primarily **religious leaders** (Christian, Muslim, other non-witchcraft religions).
12. Advocacy strategies that target primarily **healthcare providers**.
13. Advocacy strategies that target primarily **teachers** (in schools).
14. Advocacy strategies that target primarily **potential employers** of persons with albinism (e.g., educate employers about how technology and workplace modifications can support those with albinism)

| **POLICY INITIATIVES** |
| --- |

**HEALTH and SOCIAL POLICY**

1. Collect more accurate data (e.g., census, education, health data) on populations with albinism to improve planning and government response.
2. Develop and implement policy that creates access to (community-based) **primary healthcare**.
3. Develop and implement policy that ensures **free access to sunscreen, eye assessments, and skin cancer screening** for persons with albinism.
4. Develop and implement policy that ensures access to **specialty low vision care** (i.e., optometry or ophthalmology).
5. Develop and implement national policy to **fund health and disability research** on albinism.
6. Develop and implement policy targeted at **school curriculum** (primary, secondary, university) that dispels stereotypes about albinism.
7. Develop and implement policy that ensures access to education for persons with albinism in **integrated classrooms**.
8. Develop and implement policy that provides appropriate and **disability-specific education supports** and instruction by qualified professionals.
9. Develop and implement **poverty reduction** strategies.
10. Develop and implement policy specific to **older persons with albinism**.

**PROFESSIONAL GROUPS**

1. Develop and implement policy for the creation of the role of a **National Officer for Albinism** (within a human rights framework).
2. Develop and implement policy for the creation the role of a salaried **Local Officer or Advocate for Albinism** in every district where human rights are particularly compromised (e.g., through targeted attacks).
3. Develop and implement policy for **healthcare curriculum** about albinism, healthcare, and human rights.
4. Develop and implement policy to provide sufficient resources to increase the number of **teaching assistants**, particularly in primary schools (having an assistant ensures students have accessible materials and training in technology use in the early years).
5. Develop and implement policy to ensure resourcing to increase the number of **vision specialist teachers** trained in visual impairment (TVI).
6. Develop and implement policy for equipping teachers for **classroom modifications** (e.g., seating, lighting) for inclusive education for children with albinism.
7. Develop and implement policy to ensure **disability insurance agents** are familiar with albinism to support fair decisions (e.g., with the specific vision disabilities).

**LEGISLATION AND REGULATION** (as forms of policy)

1. Develop **Regional Action Plans** for a comprehensive, intersectoral approach to human rights and albinism.
2. Develop **non-discrimination legislation** in each country for the protection of persons with albinism, including laws against colourism.
3. Create legislation to mandate **equal employment opportunity** for persons with albinism.
4. Create legislation to **mandate accessibility standards for employment** (e.g., to guarantee the type of jobs that persons with albinism can be hired into, for example, jobs with prolonged sun exposure would not be allowed).
5. Develop **Child Protection Policies** and services across all social sectors – especially social welfare, education, health, security and justice – to support prevention and response to trauma, violence, and exploitation of children with albinism.
6. Develop, implement or revise **Witchcraft Acts** (laws preventing or restricting the practice of witchcraft).
7. Develop legislation and policies for the **regulation of witchcraft practitioners** and traditional healers.
8. Develop and implement policy for states to ensure **counseling and** **compensation for victims** (and families) who have suffered harm, death or discrimination due to the condition of albinism.
9. Develop and implement regulation that **bans TV or radio programs that stereotype** persons with albinism
10. Develop and implement policies for the **regulation of Nollywood** that perpetuate myths, stereotypes, and stigma about persons with albinism.

**POLICING**

1. Develop and implement policies in order to pursue justice at the highest international level (e.g., prosecute violence against persons with albinism in **international courts**).
2. Establish an effective **special prosecution task force** to investigate, prosecute and punish the perpetrators and masterminds of trafficking of body parts of persons with albinism.
3. Develop policies to **strengthen local policing** to ensure local protection for persons with albinism
4. Develop **sting operations** to intercept the trafficking of body parts (similar to operations used to target trafficking of animal parts in Africa).

| **RESEARCH TOPICS** |
| --- |

**STIGMA, BELIEFS, AND MYTHS**

1. Research on discrimination against persons with albinism **in various sectors** (i.e., political, medical, vocational, and educational settings).
2. Research on how stigma about persons with albinism takes various forms in **different geographic locales**.
3. Research on the impact of intersecting **global and local moralities** on persons with albinism (e.g. value systems).
4. Research on dynamics of the **visibility** of albinism (e.g., representation in the media of persons with albinism as “other”).
5. Research on the **experience of older persons** with albinism (e.g., how ageism intersects with access to health and social care and other forms of discrimination).
6. Research on the myth (i.e., known as the **‘death myth’**) that people with albinism do not die normal deaths, but disappear at the end of their lives
7. Research on how to **demystify stigma and end discrimination** against persons with albinism.
8. Research on **ways to dismantle myths** around the nature of albinism that relate to public health, including the idea that albinism is contagious.
9. Research on the right to inclusion, and **how to create more inclusive environments** for persons with albinism.

**PSYCHOLOGICAL WELLBEING**

1. Research on how persons with albinism may **internalize social stigma**.
2. Research on **resilience** (e.g., presence, facilitators, barriers) in persons with albinism and their families.
3. Research on the **impact of trauma** (e.g., violence, threats) on persons with albinism and their families.
4. Research on **trauma-informed care and policy** for persons with albinism and their families.
5. Research on **social change in Africa**, including demographic trends, and how these relate to the security and wellbeing of persons with albinism.

**HEALTH AND SOCIAL CARE**

1. Research on **epidemiology** (e.g. prevalence, how the distribution of albinism is varied amongst countries, for example appears higher in Africa).
2. Research on **access to general healthcare** (not albinism-specific needs like vision and skin care) for persons with albinism.
3. Research on **genetic testing** for persons with albinism, including issues of access.
4. Research on how **vision impairment** impedes education, confidence, independence and social inclusion.
5. Research on how **vision rehabilitation** can empower and provide access to education.
6. Research on how to enhance access to **locally-based specialist low vision care** **and equipment** for persons with albinism.
7. Research on how **cultural views of wearing spectacles** can impede the successful rehabilitation of persons with albinism.
8. Research on the impact of **cultural, religious and traditional beliefs** on access to health for persons with albinism.
9. Research on how **humanitarianism** impacts persons with albinism
10. Research on ways to improve **healthcare providers’ knowledge and practices** around caring for persons with albinism.
11. Research on **how health and social services can be integrated** with each other as well as with other services (e.g. policing, education, or transportation) to create supportive and inclusive environments for persons with albinism and their families.
12. Research on **social support** provided for persons with albinism (e.g. reviewing existing resources; community responsibility, and intersectoral approaches).

**FAMILY**

1. Research on the **human right to a family life** for families with children with albinism.
2. Research on the **human right for child protection** for children with albinism (e.g. sheltered accommodation; long-term solutions).
3. Research on **children** with albinism (e.g. their perspective within a social-relational model of disability) to understand their lives and the barriers they encounter.
4. Research on **families’ knowledge** of genetics (e.g. family history) and the cause and prevalence of albinism.
5. Research on the impact on and **experience of families** preventing their children with albinism from going to school because they fear they will be abducted.
6. Research on **how families** with children with albinism **can be supported** (e.g., support systems in the community, networks) for developing an acceptance and resilience for their children.

**ECONOMIC AND SOCIAL RIGHTS**

1. Research on **translating human rights** to the local context.
2. Research on how human rights violations perpetrated on people with albinism **intersect** with other forms of oppression.
3. Research on how the rapid shift from local, **agrarian economies to global market economies** relates to the security and wellbeing of persons with albinism.
4. Research on **how poverty impacts** persons with albinism, and how this can be managed
5. Research on the **variability of service** provided for persons with albinism across rural and urban locations (e.g. regional disparities).
6. Research to identify factors that impede persons with albinism from realizing their **right to education**.
7. Research on necessary supports for **accommodating students** with albinism in the classroom.
8. Research on **access to suitable employment** for persons with albinism.
9. Research on **accommodations** for people with albinism **in the workplace**.
10. Research on **educating employers** about albinism, with a view to increasing inclusion of persons with albinism in the workplace.

**RELIGION AND CHURCH**

1. Research on **everyday religion** in Africa, and how this relates to the security and wellbeing of persons with albinism.
2. Research on **African ontologies** (philosophies and worldviews) and their relation to how persons with albinism are viewed.
3. Research on the **role of faith communities** in supporting persons with albinism (including in addressing witchcraft accusations, countering stigmatization and fear, or offering counseling).
4. Research on **how to engage faith/spiritual leaders** to combat prejudice and violence against persons with albinism.

**WITCHCRAFT**

1. Research on **the interplay between cultural rights and non-discrimination** (e.g., the right to hold witchcraft-based beliefs while persons with albinism have the right to non-discrimination).
2. Research on the creation of an **inventory of harmful cultural practices** in relation to albinism.
3. Research on the **human sacrifice** of persons with albinism, whether as ritual or for other motivations.
4. Research on the **impact of witchcraft beliefs and practices** on persons with albinism.
5. Research on the **incidence of ritual killings** (*muti)* of persons with albinism.
6. Research on **organized crime network**s, ‘contract killers’, and their role in human rights violations against persons with albinism.
7. Research on how the **mining and fishing industries** are implicated in the demand and supply of the body parts of persons with albinism
8. Research on how the **economies of religion** (with demand/supply models, and competition for consumers) relate to the *muti* murder of persons with albinism.
9. Research on how **monetary gain and market values** relate to witchcraft, *muti*, and the trade of body parts of persons with albinism.
10. Research on the potential link between **elections and an increased incidence of attacks** on persons with albinism
11. Research on **healthcare providers’ views** on witchcraft in relation to albinism.
12. Research on how **healthcare professionals interact** with traditional healers, including witch doctors.
13. Research on the **factors that lead witch doctors to spread beliefs** about the ‘special powers’ of persons with albinism.
14. Research on **the best ways to engage with witch doctors** in changing their practices affecting persons with albinism.
15. Research on **community** **responses to ritual killings** (*muti)* of persons with albinism.
16. Research on **government responses** (e.g., legislation, policing) to ritual killings (*muti)* of persons with albinism.

**LAW**

1. Research on the **applicability of international criminal law** in cases of attacks on persons with albinism
2. Research on the relevant provisions and **gaps in international law** regarding trafficking in body parts
3. Research on **human trafficking** in relation to albinism (e.g., incidence, prevention).
4. Research on the **legislation needed to grant asylum** (refugee status) to persons with albinism (e.g., in countries like the U.S.).
5. Research on national laws, law enforcement, and police procedures in relation to **policing** to protect persons with albinism.
6. Research on the **effectiveness of shelters, refuges, or safehouses** to protect persons with albinism.
7. Research on the operationalization of the **2030 sustainable development agenda** principle of “leaving no one behind”
8. Legal research on a **specific international convention** or treaty on albinism (e.g., United Nation Convention of the Human Rights of Persons with Albinism).

**MEDIA AND COMMUNICATION**

1. Research on the ongoing **media bias** in Europe and the U.S. (e.g., the stereotype of the ‘evil albino’).
2. Research on the **impact of media** (radio, social media, news, film) to perpetuate and challenge stereotypes about persons with albinism.
3. Research on the role **Nollywood films** play in spreading myths about albinism.

**RESEARCH ABOUT ADVOCACY**

1. Research to **map networks of influence** (e.g., including civil society organizations) that contribute to the security and wellbeing of persons with albinism.
2. Research on the **effectiveness of albinism advocacy strategies** for coordinated, sustained social change.
3. Research on how to strengthen the **advocacy work of international organizations** such as the United Nations and World Health Organization
4. Research on how the relationship between human rights and albinism **compares** with the relationship between human rights and other disabilities or markers of social difference.
5. Research on how advocacy for persons with albinism could be strengthened by **learnings from human rights-based interventions** undertaken to support other marginalized populations.
6. Research on **how to integrate the voices** of persons with albinism to affect social change.
7. Research on the **social dynamics with and within the disability movement** (internationally, nationally, and locally) that impact advocacy and policy initiatives on albinism.
8. Research on how to **build research teams** that integrate local and global resources and expertise on albinism.
